# PhyloGibbs: A Gibbs Sampling Motif Finder That Incorporates Phylogeny

**DOI:** 10.1371/journal.pcbi.0010067

**Published:** 2005-12-09

**Authors:** Rahul Siddharthan, Eric D Siggia, Erik van Nimwegen

**Affiliations:** 1 Center for Studies in Physics and Biology, The Rockefeller University, New York, New York, United States of America; 2 Institute of Mathematical Sciences, Taramani, Chennai, India; 3 Division of Bioinformatics, Biozentrum, University of Basel, Basel, Switzerland; University of California at San Diego, United States of America

## Abstract

A central problem in the bioinformatics of gene regulation is to find the binding sites for regulatory proteins. One of the most promising approaches toward identifying these short and fuzzy sequence patterns is the comparative analysis of orthologous intergenic regions of related species. This analysis is complicated by various factors. First, one needs to take the phylogenetic relationship between the species into account in order to distinguish conservation that is due to the occurrence of functional sites from spurious conservation that is due to evolutionary proximity. Second, one has to deal with the complexities of multiple alignments of orthologous intergenic regions, and one has to consider the possibility that functional sites may occur outside of conserved segments. Here we present a new motif sampling algorithm, PhyloGibbs, that runs on arbitrary collections of multiple local sequence alignments of orthologous sequences. The algorithm searches over all ways in which an arbitrary number of binding sites for an arbitrary number of transcription factors (TFs) can be assigned to the multiple sequence alignments. These binding site configurations are scored by a Bayesian probabilistic model that treats aligned sequences by a model for the evolution of binding sites and “background” intergenic DNA. This model takes the phylogenetic relationship between the species in the alignment explicitly into account. The algorithm uses simulated annealing and Monte Carlo Markov-chain sampling to rigorously assign posterior probabilities to all the binding sites that it reports. In tests on synthetic data and real data from five *Saccharomyces* species our algorithm performs significantly better than four other motif-finding algorithms, including algorithms that also take phylogeny into account. Our results also show that, in contrast to the other algorithms, PhyloGibbs can make realistic estimates of the reliability of its predictions. Our tests suggest that, running on the five-species multiple alignment of a single gene's upstream region, PhyloGibbs on average recovers over 50% of all binding sites in *S. cerevisiae* at a specificity of about 50%, and 33% of all binding sites at a specificity of about 85%. We also tested PhyloGibbs on collections of multiple alignments of intergenic regions that were recently annotated, based on ChIP-on-chip data, to contain binding sites for the same TF. We compared PhyloGibbs's results with the previous analysis of these data using six other motif-finding algorithms. For 16 of 21 TFs for which all other motif-finding methods failed to find a significant motif, PhyloGibbs did recover a motif that matches the literature consensus. In 11 cases where there was disagreement in the results we compiled lists of known target genes from the literature, and found that running PhyloGibbs on their regulatory regions yielded a binding motif matching the literature consensus in all but one of the cases. Interestingly, these literature gene lists had little overlap with the targets annotated based on the ChIP-on-chip data. The PhyloGibbs code can be downloaded from http://www.biozentrum.unibas.ch/~nimwegen/cgi-bin/phylogibbs.cgi or http://www.imsc.res.in/~rsidd/phylogibbs. The full set of predicted sites from our tests on yeast are available at http://www.swissregulon.unibas.ch.

## Synopsis

Computational discovery of regulatory sites in intergenic DNA is one of the central problems in bioinformatics. Up until recently motif finders would typically take one of the following two general approaches. Given a known set of co-regulated genes, one searches their promoter regions for significantly overrepresented sequence motifs. Alternatively, in a “phylogenetic footprinting” approach one searches multiple alignments of orthologous intergenic regions for short segments that are significantly more conserved than expected based on the phylogeny of the species.

In this work the authors present an algorithm, PhyloGibbs, that combines these two approaches into one integrated Bayesian framework. The algorithm searches over all ways in which an arbitrary number of binding sites for an arbitrary number of transcription factors can be assigned to arbitrary collections of multiple sequence alignments while taking into account the phylogenetic relations between the sequences.

The authors perform a number of tests on synthetic data and real data from Saccharomyces genomes in which PhyloGibbs significantly outperforms other existing methods. Finally, a novel anneal-and-track strategy allows PhyloGibbs to make accurate estimates of the reliability of its predictions.

## Introduction

Transcription factors (TFs) are proteins that bind in a sequence-specific manner to short DNA segments (“binding sites”), most commonly in intergenic DNA upstream of a gene, to activate or suppress gene transcription. Their DNA-binding domains recognize collections of short related DNA sequences (“motifs”). One generally finds that, although there is no unique combination of bases that is shared by all binding sites, and although different bases can occur at each position, there are clear biases in the distribution of bases that occur at each position of the binding sites. A common mathematical representation of a motif that takes this variability into account is a so-called weight matrix (WM) [1,2] *w,* whose components *w*_α*i*_ give the probabilities of finding base α ∈ {A, C, G, T} at position *i* of a binding site. The main assumption underlying this mathematical representation is that the bases occurring at different positions of the binding site are probabilistically independent. This in turn follows, under some conditions [3], from the assumption that the binding energy of the protein to the DNA is a sum of pairwise contact energies between the individual nucleotides and the protein.

There are several algorithms that are based on the WM representation that detect, ab initio, binding sites for a common TF in a collection of DNA sequences [4–7]. These algorithms broadly fall into two classes. One class, of which MEME [6] is the typical representative, searches the space of all WMs for the WM that can best explain the observed sequences. The class of “Gibbs sampling” algorithms, of which the Gibbs motif sampler [4,5] is the typical representative, instead samples the space of all multiple alignments of small sequence segments in search of the one that is most likely to consist of samples from a common WM.

A crucial factor for the success of ab initio methods is the ratio of the number of binding sites to the total amount of DNA in the collection of sequences. That is, the larger the number of binding sites in the set, and the smaller the total amount of DNA, the more likely it is that ab initio methods can discover the binding sites among the other DNA sequences. In order to ensure a reasonable chance of success one thus needs to provide these methods with collections of sequences that are highly enriched with binding sites for a common TF. One possibility is to use sets of upstream regions from genes that appear co-regulated in microarray experiments (e.g., [8,9]) or that were bound by a common TF in ChIP-on-chip experiments (e.g., [10]). Another possibility is to use upstream regions of orthologous genes from related organisms. Here the assumption is that (most of) the regulation of the ancestor gene, and thus its binding sites, has been conserved in the orthologs that descend from it.

This latter approach is in general complicated by a number of factors. When searching for regulatory sites in sequences that are not phylogenetically related, such as upstream regions of different genes from the same organism, one may simply look for short sequence motifs that are overrepresented among the input sequences. If the set of species from which the orthologous sequences derive are sufficiently diverged, one may simply choose to ignore the phylogenetic relationship between the sequences and treat the orthologous sequences in the same way as sequences that are not phylogenetically related. This was, for instance, the approach taken by McCue et al. [11,12], where the Gibbs motif sampler algorithm [4,5] was used on upstream regions of proteo-γ bacteria.

However, this approach is not applicable to datasets containing more closely related species, where some of the sequences will exhibit significant amounts of similarity simply because of their evolutionary proximity. Moreover, the amount of similarity will depend on the phylogenetic distance between the species, and it is clear that finding conserved sequence motifs between orthologous sequences from closely related species is much less indicative of function than finding sequence motifs that are conserved between distant species. One will in general thus have to distinguish conservation due to functional constraints from conservation due to evolutionary proximity, and to do this correctly, the phylogenetic relationship between the sequences has to be taken into account.

A second challenge in using orthologous intergenic sequences from multiple species is the nontrivial structure of their multiple alignments. One typically finds a very heterogeneous pattern of conservation: well-conserved blocks of different sizes and covering different subsets of the species are interspersed with sequence segments that show little similarity with the sequences of the other species.

The technique of “phylogenetic footprinting” (e.g., [13–17]), restricts attention to only those sequence segments in the genome of interest that show significant conservation with the other species. The conserved regions for multiple genes are then searched for common motifs by a variety of techniques. It is unclear, however, to what extent regulatory sites are restricted to such conserved segments. For instance, several studies of *Drosophila* and yeast [18–20] have shown that there is no strong correlation between where experimentally annotated binding sites occur and whether that region is conserved. Thus, at least for yeast and flies, considerable information is lost by focusing on the conserved regions only.

We thus decided to retain the entire patchwork pattern of conserved sequence blocks and unaligned segments. Our strategy is implemented by a Gibbs sampling approach, and a preliminary account of the algorithm was presented in [21]. The algorithm operates on arbitrary collections of both phylogenetically related sequences, such as orthologous intergenic regions, and sequences that are not phylogenetically related, such as upstream regions of different genes from the same organism. The phylogenetically related groups of sequences in the input are pre-aligned into local multiple alignments where clearly similar sequence segments are aligned into blocks and sequence segments of no or marginal similarity are left unaligned [22]. Although the algorithm can also take global multiple alignments as input, we believe that these often force phylogenetically unrelated segments into aligned blocks. This may adversely affect the performance of the algorithm. We score putative sites within blocks of aligned sequences with an evolutionary model that takes the phylogenetic relationships of the species into account, while putative sites in unaligned segments are treated as independent occurrences. This Bayesian model defines a probability distribution over arbitrary placements of putative binding sites for multiple motifs, and we sample it with a Monte Carlo Markov chain. We first use simulated annealing to search for the globally optimal configuration of binding sites. The motifs in this configuration (which hopefully corresponds to the global optimum) are then “tracked” in a further sampling run to estimate realistic posterior probabilities for all the binding sites that the algorithm reports.

Recently a number of other algorithms have been developed that search for regulatory motifs in groups of phylogenetically related sequences. Probably the first algorithm that was proposed is a generalization of the Consensus algorithm [7] called PhyloCon [23]. PhyloCon operates on sets of co-regulated genes and their orthologs. It is a greedy algorithm that first finds ungapped alignments of similar sequence segments in sets of orthologous sequences, and then combines these alignments from different upstream regions into larger alignments. This algorithm does not take any phylogenetic information into account, i.e., closely related sequences are treated the same as distantly related sequences. Other drawbacks of this algorithm are that it assumes that each motif will have exactly one site in each of the intergenic regions and that it assumes that this site is conserved in all orthologs.

More closely related to PhyloGibbs's approach are two recent algorithms [24,25] that generalize MEME [6] to take the phylogenetic relationships between species into account. The main difference between EMnEM and PhyME is that PhyME uses the same evolutionary model for the evolution of binding sites as PhyloGibbs, which takes into account that binding sites evolve under constraints set by a WM, whereas EMnEM simply assumes an overall slower rate of evolution in binding sites than in background sequences. Another difference is that PhyME, like PhyloGibbs, treats the multiple alignment more flexibly than EMnEM, which demands a global multiple alignment. The main difference between PhyloGibbs and these algorithms is of course that PhyloGibbs takes a motif sampling approach, which allows us to search for multiple motifs in parallel, whereas PhyME and EMnEM use expectation maximization (EM) to search for one WM at a time.

In the following sections, we first describe our Bayesian model that assigns a posterior probability to each configuration of binding sites for multiple motifs assigned to the input sequences. We start by describing the model for phylogenetically unrelated sequences, which is essentially equivalent to the model used in the Gibbs motif sampler [4,5], and then describe how this model is extended to datasets that contain phylogenetically related sequences. After that we describe the move set with which we search the state space of all possible configurations, and the annealing and “tracking” strategy that we use to identify the significant groups of sites.

We then present examples of the performance of ours and other algorithms on both synthetic and real data. The synthetic datasets consist of mixtures of WM samples and random sequences, which is in accordance with assumptions that all algorithms make. This allows us to compare the performance of the algorithms in an idealized situation that does not contain the complexities of real data. These tests also show to what extent binding sites can be recovered for this idealized data as a function of the quality of the WMs, the number of sites available, and the number of species available and their phylogenetic distances. For our tests on real data we use 200 upstream regions from *Saccharomyces cerevisiae* that have known binding sites from the collection [26], and compare the ability of the different algorithms to recover these sites when running on multiple alignments of the orthologs of these upstream regions from recently sequenced *Saccharomyces* genomes [15,16]. Finally, we run PhyloGibbs on collections of upstream region alignments that were annotated in [27] to contain binding sites for a common TF based on data from ChIP-on-chip experiments, and we extensively compare PhyloGibbs' results with the annotations in [27] and with the literature.

## Results

### Model for Phylogenetically Unrelated Sequences

In order to motivate and explain our model for phylogenetically related sequences it is helpful to first introduce the model for sequences that are not phylogenetically related. In this context, “not phylogenetically related” means that for any pair of sequences in the input data, their common ancestor sequence is sufficiently far in the evolutionary past that mutations have been introduced multiple times at each position in the sequences. That is, any similarity left between the input sequences cannot be due to evolutionary proximity.

**Figure 1:**
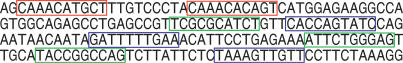
Binding Site Configuration A window, in our terminology, is a possible binding site for a TF; in the case of phylogenetically unrelated sequences it is simply a set of *m* contiguous bases in a sequence, with *m* the binding site width. This figure shows a configuration *C* containing a total of eight windows (rectangles) for three different WMs (red, blue, and green). Note that a single sequence of length *L* has *L* − *m* + 1 windows in it.

We assume that our data contain an unknown number of sites for an unknown number of different TFs. The state space of possible solutions to the problem of identifying the binding sites contained in these sequences consists of all possible ways in which one can assign groups of binding sites to these sequences. An example of such binding site assignments, which we call “configurations,” is shown in Figure 1.

Assuming that the width of the binding sites is *m* bases, each contiguous segment of *m* bases in our dataset is a potential binding site. We call such *m*-base segments “windows” and will generalize this concept later for phylogenetically related sequences. We label the WMs for the different TFs with “colors” one, two, etc., and use the color zero to indicate nonfunctional “background” sequence. Thus, formally, a configuration *C* is an assignment of nonzero colors to a particular set of nonoverlapping windows. Note that the requirement that colored windows cannot overlap means that a colored window “blocks” up to 2(*m −*1) others.

Given a dataset of input sequences *S,* our model assigns to each possible configuration *C* a posterior probability *P*(*C*|*S*). Using Bayes's theorem we have


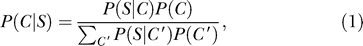


where *P*(*C*) is the prior probability of configuration *C*. As described in Materials and Methods, PhyloGibbs provides for priors that incorporate prior information on the likely number of motifs and binding sites in the input sequences. The likelihood function *P*(*S*|*C*) gives the probability that, for each color in *C,* all sequences of the same color were sampled from a common WM, multiplied by the probability of the remaining sequences under a “background” model. That is, we formally have


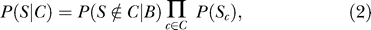


where we denote all sequence that is colored zero in the configuration *C* by *S* ∉ *C,* the background model for this sequence by *P*(*S* ∉ *C*|*B*), the set of sequences in windows with color *c* as *S_c_,* and the probability that all sequences in *S_c_* were drawn from a common WM by *P*(*S_c_*). Expressions for all these quantities are derived in Materials and Methods. The probability *P*(*S_c_*) that all sequences in color *c* were sampled from the same WM is obtained by taking the probability *P*(*S_c_*|*w*) that all sequences *S_c_* were sampled from a particular WM *w,* and integrating this over all possible WMs *w* [28].

### Incorporating Phylogeny

When considering datasets that contain phylogenetically related sequences, such as orthologous intergenic regions from related species, the main problem is distinguishing sequence similarity that is due to evolutionary proximity from sequence similarity that arises from functional constraints. That is, when calculating the probability *P*(*S*) that a group of sequences *S* are all binding sites for a common WM, we should treat sequences that are orthologous by a different model than those that are not phylogenetically related. We derive such a model below. In order to apply this model we also have to determine which parts of the input sequences are orthologous, i.e., we need to provide a multiple alignment. We could in principle let the algorithm search both the space of multiple alignments and the space of binding site configurations *C* at the same time, but we decided that this state space is likely too large to search effectively. Moreover, for closely related species large segments of the orthologous intergenic regions can be unambiguously aligned, and by pre-aligning these we significantly reduce the search space for the algorithm.

Our strategy is thus to first produce a multiple alignment and then search the space of binding site configurations that are consistent with this alignment. Standard global multiple alignment algorithms [29–31] can be used for this task, and PhyloGibbs can be run on the outputs they produce. However, as discussed in the Introduction, alignments of orthologous intergenic regions from related species (such as the recently sequenced *Saccharomyces* species [15,16]) show a mosaic pattern of well-conserved blocks interspersed with stretches of unconserved sequence, and global alignment algorithms may spuriously align many of these phylogenetically unrelated parts. Binding sites are typically not restricted to conserved regions [18,19], and spurious alignment of phylogenetically unrelated regions may hamper the discovery of binding sites contained within them. Our strategy is thus to only align those parts of the intergenic regions that are clearly orthologous and can be unambiguously aligned, and to leave the rest unaligned. In searching binding site configurations we demand consistency of the configuration with the aligned orthologous blocks, but at the same time also allow windows to be placed in unaligned parts of the sequences. Such syntenic local multiple alignments are produced by the algorithm Dialign [22], and we used this algorithm for producing the multiple alignments of the datasets that we report on below (see [32] for a recent review of the performance of various alignment algorithms on orthologous intergenic DNA). Another algorithm that produces syntenic local multiple alignments and is especially suited for large genomic regions is Threaded Blockset Aligner [33].

**Figure 2:**
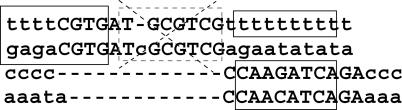
An Alignment of Four Sequences Showing Three Legitimate Windows and One Illegitimate Window Vertically aligned capital letters are phylogenetically related bases, assumed to have evolved from a common ancestor. Thus, any window placed on these bases is extended to cover all related bases. Three legitimate windows are surrounded by solid boxes. The window surrounded by the dotted box is illegitimate because the gap in the top sequence makes the alignment of bases inconsistent. Note that lower case letters are not aligned and that, in order to complete a window with aligned sequences, one may slide lowercase bases “through” adjoining gaps. For example, if the window on the bottom two sequences were to move two steps to the left, the “c” and “a” on the left side of the preceding gaps would slide through the gaps to the right to complete the window.

Once we have a syntenic multiple local alignment, we treat columns of aligned bases as phylogenetically related, i.e., arising from a common ancestor base. The state space again consists of all possible configurations of binding sites but now with the constraint that “windows” that include aligned bases have to extend over all sequences in the alignment. That is, we assume that if a binding site occurs in a sequence segment that is aligned with sequence segments from the other species, then binding sites for the same TF have to occur in the corresponding positions of these other sequence segments. To this end we extend the concept “window” (denoting a position of a potential binding site) to multiple local alignments, as illustrated in Figure 2.

The figure shows a sample stretch of four aligned sequences, where uppercase letters are aligned and lowercase letters are “independent.” In an initial pass the program identifies the set of all legitimate “windows” in the entire sequence data. Each of these windows may encompass one or more sequences. The windows must contain consistently aligned uppercase letters: there should not be “gaps” that give inconsistent spacing between aligned uppercase letters. For example, in Figure 2, the sequences boxed with solid lines show consistent windows, whereas a dotted line boxes an inconsistent window. Note that, as in the leftmost window in the figure, lowercase letters can be used to complete a window in which only some letters are uppercase. The details of identifying the set of all legitimate windows in the sequence data are described in Materials and Methods. At the end of this procedure, we have a list of windows that represent potential sites for TF binding sites; some of these windows contain only one sequence and represent a potential independent occurrence of a binding site, while others contain multiple sequences and represent potential binding sites that evolved from a common ancestor site and were conserved across multiple species.

Next, we need to generalize our probabilistic model to multiply aligned orthologous sequences. For the single-sequence windows of the previous section, the probability *P*(*s*|*w*) of observing the sequence *s* given a WM *w* is simply given by


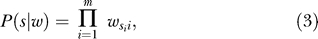


with *s_i_* the base at position *i* of *s*. For a window overlaying a region of multiply aligned sequences, the single base *s_i_* is replaced by the set of bases *W_i_* in the *i*th column of the alignment. We now replace the probability 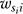 with the probability *P*(*W_i_*|*w*) that the bases *W_i_* in the *i*th column of the window derive from a common ancestor base, and have been evolving under the selective constraints set by the requirement that they remain binding sites for the TF represented by WM *w*. Our evolutionary model assumes that mutations are introduced at a fixed rate and that the probability for selection to fix a mutation toward base α in the *i*th column of a site is proportional to the WM entry *w*_α*i*_. Since the positions are mutually independent we have for the whole window


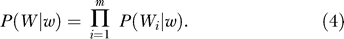


The probability *P*(*S_c_*|*w*) that all windows *W* ∈ *c* in color *c* evolved under the constraints set by WM *w* is simply given by the product


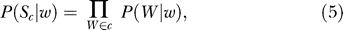


and the probability *P*(*S_c_*) is again given by an integral of *P*(*S_c_*|*w*) over the space of all possible WMs. The background score *P*(*W*|*B*) for a window *W* is given by the exact same expression as *P*(*W*|*w*) except that the WM entries *w*_α*i*_ are replaced with the background probabilities for the bases in each column. Detailed derivations and explicit expressions are provided in Materials and Methods.

### Move Set

The last two sections have explained the posterior probability *P*(*C*|*S*) that we assign to configurations of binding sites *C* given the input data *S*. PhyloGibbs samples the space of all possible configurations *C* using a Monte Carlo Markov-chain sampling strategy [34] using a move set with a number of different moves. Generally, the moves in our move set operate on configurations either by shifting one or more colored windows in the configuration, or by adding/removing a colored window to/from the configuration. Formally, a “move” consists of taking the current configuration *C,* constructing the set *X* of all configurations *C′* ∈ *X* that differ from *C* by a “single change” (e.g., moving the position of a single colored window), and then choosing one of these configurations *C′* according to the distribution 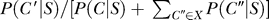 . In order for the repeated application of these moves to sample the whole space of configurations in proportion to their probabilities *P*(*C*|*S*), two conditions are sufficient. First, the move set needs to be “ergodic”: each configuration *C* must be reachable by repeated application of the moves in the move set. And second, we demand that “detailed balance” be satisfied: for any pair of states *C* and *C′* the probabilities *P*(*C* → *C′*) of moving from *C* to *C′* and *P*(*C′* → *C*) of moving from *C′* to *C* under any of the moves must satisfy


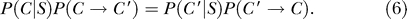


The most important of the moves in our move set is the “window shift” move, which takes a single window and resamples its position. Since this type of move is generally referred to as Gibbs sampling, i.e., one samples a joint probability distribution by resampling one variable of the joint distribution at each time step while keeping the other variables fixed, and because of the similarities with the original Gibbs motif sampling algorithm [4,5], we have called our algorithm PhyloGibbs. Other moves in our move set include picking a window at random and recoloring it, and shifting all windows in a color by the same amount. Each time step of the algorithm corresponds to a “cycle” of a fixed number of moves of each type. The moves in our move set were designed to avoid the algorithm getting trapped in local maxima, to speed up convergence, and to ensure ergodicity. They are described in detail in Materials and Methods.

### Strategy: Anneal and Track

By repeating moves from the move set described in the previous section PhyloGibbs will, in the limit of long time, sample each configuration *C* according to its posterior probability *P*(*C*|*S*). Even though the distribution *P*(*C*|*S*) represents everything that can be inferred from the data using our model, we still have to decide how to usefully summarize this information. There is, to our knowledge, currently no generally satisfactory solution to this problem (see the discussion in [28]). One would like the summary to report all the relevant features that are shared by the configurations *C* with high posterior probability *P*(*C*|*S*). In particular, one would like to identify groups of windows that with high probability share a color. Given a reference set of windows, it is straightforward to check what fraction of the time different subsets from this reference set are colored the same, and the fraction of the time that other windows are co-colored with windows in this reference set. However, one obviously cannot check this for all possible subsets of windows, and we thus have to decide what reference sets we want to “track.”

Our current strategy is to first search for the configuration C* that has the globally maximal posterior probability *P*(*C*|*S*) and to take the sets of co-colored windows in this configuration as the reference sets. We use simulated annealing to search for this globally optimal configuration C*, which we call the “reference configuration.” Instead of sampling configurations from the distribution *P*(*C*|*S*) we raise each probability to the power β and sample from the posterior *P̃*(*C*|*S*) ∝ *P*(*C*|*S*)^β^. Initially we set β = 1 and slowly increase β with time until the sampler “freezes” into a configuration C* with (at least locally) maximal probability *P*(C*|*S*).

The annealing phase is followed by a tracking phase in which we sample from the distribution *P*(*C*|*S*) in order to estimate, for each window *w* and each color *c,* the probability *p*(*w,c*) that window *w* belongs to the same motif (color) as the windows in color *c* of the reference configuration C*. The probabilities *p*(*w,c*) are estimated as follows. After every cycle of moves we compare the current configuration *C* with the reference configuration C*. Specifically, for every color *c* in the reference configuration C* we determine which color c̃ of the current configuration *C* matches *c* most closely (allowing for shift and reverse-complement of the windows in c̃ with respect to the windows in *c;* see Materials and Methods). For every window *w* in c̃ (properly shifted to align with *c*), we add a count of one to the number of times *n*(*w,c*) that *w* is associated with color *c*. At the end we set *p*(*w,c*) = *n*(*w,c*)/*n,* with *n* the total number of cycles.

In its default mode of operation PhyloGibbs reports the following results for the input set of sequences *S:* (1) the configuration C* that has maximal posterior probability *P*(*C*|*S*), (2) an inferred WM for each color *c* in configuration C*, (3) for each color *c,* all windows *w* for which *p*(*w,c*) ≥ *p*_min_, with *p*_min_ a cutoff that the user can specify, and (4) a WM for each color *c* from reference configuration C* that is inferred by weighing the member windows *w* with their membership probabilities *p*(*w,c*).

Note that, in general, different member windows *w* of color *c* in reference configuration C* will have very different posterior probabilities *p*(*w,c*) to be members of the motif. In addition, for each color *c* the tracking will typically uncover windows *w* that were not a member of color *c* in configuration C* but that with reasonable probability *p*(*w,c*) belong to the motif as well. One inherent limitation of our anneal-and-track strategy is that it can in principle miss a group of windows that are co-colored a significant fraction of the time in *P*(*C*|*S*) but that do not occur in configuration C*. Trivially, if the user allows for too few colors, some motifs may be missed. It generally does not hurt the results to overestimate the number of colors (different motifs), although it increases running time. Similarly, it is also not necessary to accurately estimate the total number of sites. If the prior overestimates the total number of sites, some spurious sites in the reference configuration will simply fail to track. Similarly, if the total number of sites is underestimated, some sites that were missed in the reference configuration will be picked up in the tracking phase. To give maximal flexibility to the user, the algorithm can also, instead of doing an anneal, be given an input reference configuration and track the motifs in this configuration.

As far as we are aware, PhyloGibbs is the only motif-finding algorithm that rigorously assigns posterior probabilities *p*(*w,c*) to the binding sites that it reports by sampling the entire space of binding site configurations. The Gibbs motif sampler [4] WGibbs samples the space of configurations while keeping track of the best configuration C* that it has seen, i.e., it does not use annealing. It also reports posterior probabilities for the sites it reports, but these are based on sampling of only a small subspace of configurations around the configuration C*. Algorithms that search the space of WMs [6,25,24] use EM to find an optimal WM explaining the data and then assign posterior probabilities to reported sites by assuming this WM is correct. That is, they do not take into account the (likely) possibility that the WM found through EM is not entirely correct.

As we show below, only the rigorous sampling of the space of all configurations as implemented in PhyloGibbs is capable of assigning realistic posterior probabilities to the sites it reports. It is sometimes attempted to identify “significant” motifs by simply rerunning one or several different motif-finding algorithms and looking for recurring motifs. However, this merely generates the subsidiary problem of clustering the multiple predictions. Instead of using ad hoc scoring schemes for clustering, reported binding sites should ideally be clustered using the same probabilistic scoring that generated them, i.e., as in [28]. PhyloGibbs generalizes the binding site clustering algorithm of [28] and subsumes any problems of clustering.

### Performance on Synthetic Data: Anneal

In general there are three qualitatively different issues that contribute to the performance of motif-finding algorithms on real data. First, all motif-finding algorithms make assumptions about the data that will ignore at least some of the complexities of real data. The performance of a given motif-finding algorithm will depend on the extent to which these ignored complexities affect the algorithm's ability to perform its task. Second, the search spaces of all possible WMs or all possible binding site configurations are too large to search exhaustively, and therefore all algorithms employ heuristic methods to search for the globally optimal WMs or configurations. The extent to which the heuristic methods succeed or fail will also affect the performance of the algorithms. Third, even if the data adhere to all assumptions that an algorithm makes, and the algorithm successfully finds the global optimum in the search space, this still does not guarantee that the algorithm will recover the correct motifs and sites. That is, if the motifs are fuzzy and the sites are embedded in long background sequences it might occur that, by chance, the background contains sets of sites that are more conserved and more similar than the embedded sites. In this case it will be impossible for any algorithm to recover the true sites.

By generating synthetic data to accord, as much as possible, with the assumptions that the motif-finding algorithms make, we can study the second and third issues separately from the first issue. In this section and the next we do a number of such tests. In our first test we want to evaluate to what extent PhyloGibbs can recover a fixed number of sites embedded in a perfect alignment of orthologous sequences as the quality of the WMs and the phylogenetic distances of the orthologs are varied. At the same time, we want to test how well PhyloGibbs performs when operating on perfect alignments compared to algorithms that do not take phylogenetic information into account and that cannot operate on multiple alignments (including PhyloGibbs in the mode where it ignores phylogenetic information). This test will indicate how much performance can be improved by using phylogenetic information and multiple alignments in an ideal situation. For ease of reference, from now on we refer to all algorithms that use phylogenetic information and that can operate on multiple alignments as “phylo” algorithms, while referring to algorithms that treat all sequences as independent as “non-phylo” algorithms.

For our first test we generated synthetic datasets as follows. (1) We first generated a WM of width *w*. For each position in the WM we picked a random “consensus” base, set the probability of that base to *p,* and set the probabilities of the other bases to (1 − *p*)/3. The WM was thus parametrized by *p,* which we call its “polarization.” For some datasets we also generated random WMs by picking, for each position, a random distribution (*w_a_, w_c_, w_g_, w_t_*) uniformly from the simplex 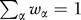 . Note that for these random WMs the “polarization” varies from column to column. (2) We sampled *s* sites from the WM and embedded them in a random sequence of length *L*. This sequence formed the ancestor sequence. (3) We then generated *S* descendant sequences as follows. For each base the descendant's base was equal to the ancestor's base with probability *q*. With probability 1 − *q* we mutated the base. Outside binding sites, a mutation replaced the ancestor base with a randomly chosen base. Within binding sites, a mutation replaced the ancestor base with a new sample from the WM. Because the WM is generally biased toward particular bases this results in more conservation within binding sites than outside them. We refer to the parameter *q* as the “proximity” of the descendants to their common ancestor. (4) We measured performance by the overlap between the predicted sites in the reference configuration C* and the embedded sites. Formally, we counted the number of bases in the intersection of predicted and embedded sites and divided by the total number of bases in embedded sites (which, in these tests, equals the total number of bases in predicted sites). A performance of one thus corresponds to a perfect overlap of the embedded and predicted sites.

**Figure 3:**
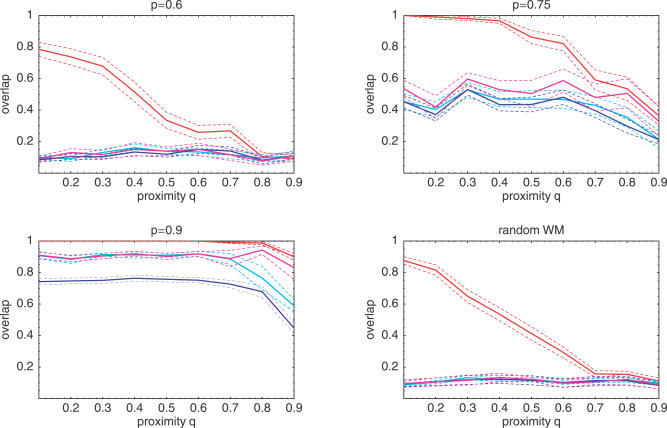
Performance of PhyloGibbs and Non-Phylo Motif-Finding Algorithms on Alignments of Orthologous Intergenic Regions as a Function of the Evolutionary Proximity of the Orthologs and the Quality of the WM PhyloGibbs with phylogeny (red), PhyloGibbs in non-phylo mode (light blue), WGibbs (dark blue), and MEME (pink) were run on alignments of *S* = 5 intergenic regions of length *L* = 500, each at a proximity *q* to the common ancestor and each containing *s* = 4 binding sites from a single WM of width *w* = 10. In the upper left panel, WMs had polarization *p* = 0.6, in the upper right *p* = 0.75, in the lower left *p* = 0.9, and in the lower right random WMs (drawn uniformly from the simplex) were used. The solid lines show the average overlaps between the predicted sites and the real sites, and the dotted lines show two standard errors (estimated from 50 different datasets generated with equal parameters for each data point).

We compared the performance of PhyloGibbs with those of non-phylo algorithms on alignments of *S* = 5 orthologous intergenic regions of length *L* = 500 containing *s* = 4 sites for a single motif, as a function of the WM polarization *p* and the proximity of the descendants *q*. The results are shown in Figure 3.

All algorithms assume that the data are a mixture of random uncorrelated background sequences and samples from a number of WMs of certain lengths. With the exception of the phylogenetic relationship of the sequences, which is ignored by the non-phylo algorithms, the synthetic data are thus in complete accordance with the assumptions that each of the algorithms make. For each algorithm we specified the correct length and number of sites. Since when using PhyloGibbs with phylogeny the windows extend over all five sequences in the alignment, we asked PhyloGibbs to predict four multi-sequence windows for a single motif, while we asked the non-phylo algorithms to search for 20 single-sequence sites for a single motif. Since for any algorithm, the performance differs substantially between input datasets that were generated with the same parameter settings, we averaged results over 50 datasets and in Figure 3 show two standard errors (dotted lines) around the average performance (solid lines).

All non-phylo algorithms, including PhyloGibbs when phylogeny is turned off, perform roughly equally well (or badly). For highly polarized WMs all non-phylo algorithms perform quite well. In contrast, for low polarizations (*p* = 0.6 in the upper left panel) or for random WMs (lower right panel), all non-phylo algorithms perform hardly better than random predictions would do (the four embedded binding sites cover 8% of the input data). The second thing to note is that, especially as phylogenetic distance between the orthologs increases (lower *q*), PhyloGibbs performs substantially better than the non-phylo algorithms. As the amount of conservation due to evolutionary proximity becomes larger than the amount of conservation due to functional constraints, i.e., as *q* becomes larger than *p,* the performance drops and the performance of PhyloGibbs becomes only marginally better than that of the non-phylo algorithms.

It is important to point out that PhyloGibbs's superior performance for these data is partly due to the fact that the five sequences have been perfectly aligned and that it is searching only through configurations that are consistent with this alignment. In contrast, the non-phylo algorithms treat the five sequences as independent and have to search a much larger space of configurations. For real data we of course do not have perfect alignments and it will generally be hard to obtain good alignments when the proximity *q* becomes small, which will negatively affect the performance of PhyloGibbs. This effect is seen below in our tests on real data.

In Figure S1 we show the results of additional tests analogous to those shown in Figure 3. In these tests we chose the WMs and phylogeny to mimic as closely as possible the situation in yeast, whose real data we study below. That is, instead of creating random WMs we used “real” WMs of yeast TFs with known binding specificities, and we used phylogenetic distances for the five descendants that are proportional to the phylogenetic distances of the *Saccharomyces* sensu stricto species that we use in our tests on real data below. The results with these more realistic synthetic datasets are quantitatively very similar to the results shown in the upper right panel of Figure 3.

It might also be asked if the non-phylo algorithms are put at a disadvantage by the fact that they have to search for a much larger number of sites. That is, to get a 50% performance PhyloGibbs needs only to get two multi-species sites correct, whereas the non-phylo algorithms need to get ten sites correct. To test this we ran MEME and WGibbs on single sequences, as opposed to groups of *S* = 5 orthologs, and asked the algorithms to find only *s* = 4 sites instead of *sS* = 20. The non-phylo algorithms MEME and WGibbs performed extremely poorly in this mode. It thus appears that, at least for this kind of synthetic data, the benefit of having more sites outweighs the negative effects of having more sequence to search through.

Although PhyloGibbs performed consistently better than the non-phylo algorithms, in many cases it recovered only a fraction of the embedded sites. Since the synthetic data were generated exactly according to the model that PhyloGibbs assumes, there are only two possible reasons for the failure of PhyloGibbs to recover the embedded sites. The first possibility is that the correct configuration, i.e., with the four binding sites occurring at the positions where they were embedded, is the globally optimal binding site configuration, but that the anneal phase failed to identify it and instead settled on an only locally optimal configuration. In that case the posterior probability *P*(*C*_cor_|*S*) of the correct configuration *C*_cor_ should be higher than the posterior probability *P*(C*|*S*) of the configuration C* that the anneal obtained. The second possibility is that the anneal did identify the globally optimal configuration, and that this configuration C* has higher posterior probability *P*(C*|*S*) than the posterior probability of the correct configuration *P*(*C*_cor_|*S*). This can happen when, by chance, positions outside of the embedded binding sites are more conserved and/or better match a common WM than the embedded sites, making it impossible for any algorithm to recover them correctly.

To investigate how often the anneal in PhyloGibbs identifies the globally optimal configuration, we compared *P*(C*|*S*) with *P*(*C*_cor_|*S*) for the runs with proximities *q* = 0.2, *q* = 0.5, and *q* = 0.8, shown in the lower right panel of Figure 3. For each value of *q* there were 50 independent datasets generated, and PhyloGibbs was run five times on each dataset. Thus, for each value of *q* there were 250 runs in total. We found that *P*(C*|*S*) ≥ *P*(*C*_cor_|*S*) for 245 of the 250 runs for *q* = 0.2, for 243 of the 250 for *q =* 0.5, and for all 250 runs for *q* = 0.8. Thus, for 98.4% of the runs, the posterior probability of the configuration found in the anneal was at least as high as the posterior probability of the correct configuration.

In conclusion, these first tests with synthetic data showed that, when sites from WMs are embedded in a random ancestor sequence, and PhyloGibbs is given a perfect alignment of a set of descendants of this sequence, it performs significantly better than algorithms that treat the descendants as independent sequences. It also shows that as the similarity between sites becomes less than or equal to the similarity between orthologous sequences due to evolutionary proximity, it becomes impossible for any algorithm to accurately recover the sites.

**Figure 4:**
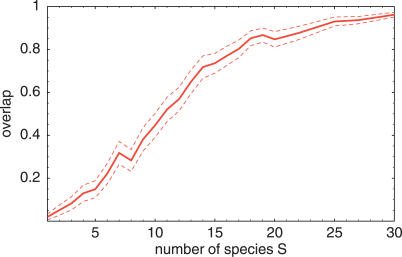
Performance of PhyloGibbs in Recovering a Single Site of a Randomly Chosen WM of Width *w* = 10 from the Alignment of *S* Orthologous Intergenic Regions of Proximity *q* = 0.5 and Length *L* = 500 as a function of *S* The solid line shows the average overlap between the true site and the predicted site and the dotted lines show two standard errors.

In the first test PhyloGibbs used both information from the overrepresentation of a motif in the data and information about the conservation of its sites. We next investigated how many species one would need, in an ideal situation, to reliably infer the location of a single binding site using conservation only. To test this we generated synthetic intergenic regions of length *L* = 500 that contained a single site for a single randomly chosen WM of width *w* = 10 and created alignments of *S* descendant sequences with proximity *q* = 0.5. We then ran PhyloGibbs for different values of *S,* asking it to search for a single multi-sequence window in the alignment. The results are shown in Figure 4.

We see that more than ten species are needed to have a 50% probability to recover a single site of a random WM at *q* = 0.5, and that as many as 25 species are necessary to recover the site with 90% probability. Note that significantly more species are necessary to recover a single site than are necessary to recover a group of multiple sites from the same WM. That is, in the lower right of Figure 3 about 40% of the quartet of sites is recovered at *q* = 0.5, whereas in Figure 4 at *S =* 5 the single site is recovered with a probability of only 0.15. In conclusion, this test has shown that if enough species are available in which a given binding site occurs, and these can be reliably aligned, then even individual sites can be reliably recovered by PhyloGibbs.

### Performance on Synthetic Data: Tracking

In the next section we compare the performance of PhyloGibbs and other algorithms that use phylogeny (PhyME [25] and EMnEM [24]) on real data from five *Saccharomyces* species. To compare and contrast with those tests we here create synthetic datasets that are comparable with those real datasets but that are idealized in the sense that they respect the assumptions that the various algorithms make about the data as much as possible. This test allows us to directly compare the performance of the phylo algorithms on idealized data, and the extent to which they outperform non-phylo algorithms in this idealized setting.

As mentioned in the previous section, our synthetic data are in accordance with all assumptions that the non-phylo algorithms make about the data, except of course for the phylogenetic relationships between the sequences. Our synthetic orthologous sequences are generated in accordance with the evolutionary model that PhyloGibbs and PhyME assume. For these two algorithms the synthetic data are thus in exact accordance with the assumptions that these algorithms make. EMnEM employs an evolutionary model that uses the same substitution matrix both within and outside of sites, but allows each position in a binding site to evolve at a different overall rate. This model is thus less realistic than the model that PhyME and PhyloGibbs use in that it ignores that the probabilities of different substitutions within a site depend on the site's WM. However, since it has more free parameters that can be fitted, we suspect that in practice it will be able to reasonably approximate the evolutionary model that PhyloGibbs and PhyME use.

**Figure 5:**
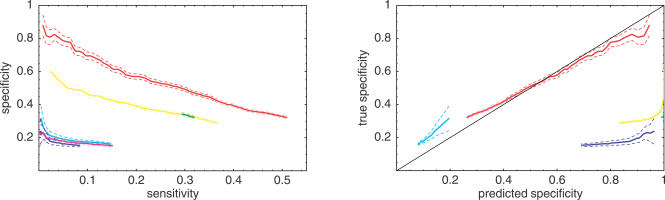
Performance of Several Motif-Finding Algorithms on Synthetic Data Prepared as for Figure 3 A total of 250 alignments of *S* = 5 orthologous intergenic regions of length *L* = 750 and proximity *q* = 0.5 were created with three binding sites sampled from each of three different random WMs. The left panel shows how the fraction of predicted sites that match true sites (specificity) depends on the fraction of true sites that are among the predictions (sensitivity) for PhyloGibbs (red), EMnEM (yellow), PhyME (green), PhyloGibbs without phylogeny (light blue), WGibbs (dark blue), and MEME (pink). Dashed lines correspond to two standard errors. The right panel shows the ability of the different algorithms to assess their own reliability. The true specificity is shown as a function of the specificity that the algorithm predicts for the sites that it reports. The black line *y* = *x* corresponds to a perfect assessment of the algorithm's reliability.

We followed the estimates of [35] and created 250 synthetic datasets, each containing nine binding sites, sampled equally from three random WMs for each intergenic region (see Figure 5 for details). We assessed the results with a plot of specificity (fraction of predictions matching true sites) versus sensitivity (fraction of true sites that were recovered). All the algorithms report posterior probabilities (or a *p*-value) for the sites they report, which we used to rank the predictions. We pooled the predictions from all datasets and then generated successively larger lists of predictions by including all predictions over a given posterior probability. For each list we then determined the specificity and sensitivity and plotted them in Figure 5 (see Materials and Methods for details).

The algorithms that ignore phylogeny did not recover more than 16% of the true sites (sensitivity), and did so with a nearly fixed specificity of around 20%, meaning that there is not much enrichment in true sites in the top versus the bottom of their ranked lists. The algorithms that exploit the phylogeny all did better for the simple reason that they operate on the perfect multiple alignments and therefore their search space is much smaller. Of these three algorithms PhyloGibbs performed best. PhyME, in common with the non-phylo algorithms, reports a very limited range of posterior probabilities for the sites it reports, which leads to a relatively small “dynamic range” in sensitivity/specificity.

Note that even with this perfectly aligned data, to get 50% of the true sites, PhyloGibbs needed to make more than twice as many predictions. Again, to determine to what extent the failure of PhyloGibbs to recover all the embedded sites was caused by the anneal getting trapped in locally optimal configurations, we compared the posterior probabilities *P*(C*|*S*) of the reference configurations with those of the correct states *P*(*C*_cor_|*S*). We found that *P*(C*|*S*) was greater than or equal to *P*(*C*_cor_|*S*) for all 250 datasets.

These synthetic data also provide the opportunity to test how well the algorithms assess their reliability, i.e., how well the reported posterior probabilities for their predictions match the specificities (fraction of predictions matching true sites) we compute by knowing the true sites. Ideally the two are the same, so that for real data one could use the posterior probabilities to gauge the fraction of correct predictions. The right panel of Figure 5 shows that with the exception of PhyloGibbs (with and without phylogeny) all algorithms were much too optimistic about the quality of their predictions: EMnEM and MEME gave posterior probabilities larger than 95% when their specificity was around 35%, and WGibbs gave posteriors of 90% when its real specificity was only 20%. MEME is not included in the right panel of Figure 5 because it reports *p*-values instead of posterior probabilities.

Both EMnEM and PhyME overestimated their specificity because they calculate their posterior probabilities for sites under the assumption that the WMs that they infer are correct. In reality, the inferred WM will often not match the true WM that generated the data. For WGibbs the overestimation stems from the restricted sampling of configurations around the one that gave the maximum posterior probability during sampling. Only PhyloGibbs bases its posterior on sampling of the whole space of binding site configurations.

In Figure S2 we present the results of a test analogous to the one in Figure 5, using again “real” WMs from yeast instead of random WMs, and using the proximities of the sensu stricto *Saccharomyces* species instead of proximity 0.5 for all descendants. All algorithms performed substantially better in these tests, which is in all likelihood mostly because of the higher information scores (see Materials and Methods) of the yeast WMs compared to random WMs. All phylo algorithms still outperformed the non-phylo algorithms, and of the phylo algorithms, PhyloGibbs performed significantly better than PhyME and EMnEM. In contrast to the test shown in Figure 5, PhyME clearly outperformed EMnEM on this test.

In summary, these tests have shown that, given perfectly aligned input sequences, all phylo algorithms substantially outperform non-phylo algorithms. The tests have also shown that, on data that are in accordance with the assumptions that the phylo algorithms make (almost all for EMnEM), PhyloGibbs outperforms the other algorithms. In addition, only PhyloGibbs is capable of reasonably estimating the reliability of its own predictions.

### Results on the Yeast Genome

To test the performance of PhyloGibbs and other algorithms on real data we decided to use data from the recently sequenced yeast genomes [15,16]. For our first test on these data we used the list of documented binding sites for yeast TFs in the *Saccharomyces cerevisiae* promoter database (SCPD) [26]. After “clean up” this list contains 466 binding sites upstream of 200 different genes with a little less than 30 bp in sites per intergenic region. Based on recent estimates [35] this probably corresponds to roughly a third of all the binding sites in these upstream regions. This dataset of experimentally verified sites allows us to quantitatively compare the abilities of the different algorithms to recover true binding sites using only the orthologous sequences of the five sensu stricto species as we did with the synthetic data in Figure 5. By comparing the performance on the real data with the performance on synthetic data we also learn about the effect, on the various algorithms, of the complexities in the real data that are not captured by the assumptions that the algorithms make.

For each of the 200 genes, we gathered its upstream region together with the orthologous upstream regions from *S. paradoxus, S. mikatae, S. bayanus,* and *S. kudriavzevii*. In complete analogy with the previous test on synthetic data, we ran PhyloGibbs with and without phylogeny, PhyME, EMnEM, WGibbs, and MEME on each of these 200 upstream regions. For PhyloGibbs we used Dialign [22] alignments of the upstream regions. PhyME uses the MLAGAN [29] software for its multiple alignments, and EMnEM uses ClustalW alignments [30]. Each of these algorithms was asked to search for three motifs and an expected three sites per motif (nine sites in total). The non-phylo algorithms were also asked to search for three motifs and to expect ten sites per motif. (We experimented with other site numbers, and ten gave the best overall results for the non-phylo algorithms.) For the phylo algorithms we also needed to specify the phylogenetic tree. We approximated the topology of the sensu stricto species by a star topology and set the branch lengths based on recent estimates of conservation rates at third positions in 4-fold degenerate codons between these genomes [35] as described in Materials and Methods.

**Figure 6:**
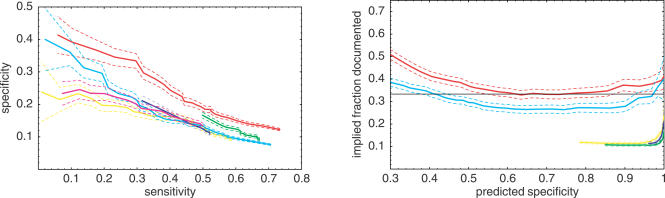
Performance of Several Motif-Finding Algorithms on 200 Alignments of Orthologous Intergenic Regions from Five *Saccharomyces* Species Containing Documented Binding Sites The left panel shows how the fraction of predicted sites that match true sites (specificity) depends on the fraction of true sites that are among the predictions (sensitivity) for PhyloGibbs (red), EMnEM (yellow), PhyME (green), PhyloGibbs without phylogeny (light blue), WGibbs (dark blue), and MEME (pink). Dashed lines correspond to one standard error. In order for the specificities, predicted by the various algorithms, to match the true specificities, we have to assume that the known sites are only a fraction of all true sites. The right panel shows what the fraction of known sites among all true sites should be in order for the algorithms' predicted specificities to match the true specificities. The black line shows an independent estimate of the fraction of real sites in these upstream regions that is documented (see text).

The left panel of Figure 6 shows the performance of the algorithms on this dataset analogously to the left panel of Figure 5. We see that, as on the synthetic data, PhyloGibbs outperformed all other algorithms on the real data. In contrast to the performances on the synthetic data, the difference between the performances of the phylo and non-phylo algorithms was much less pronounced. At very low sensitivity, PhyloGibbs run without phylogeny performed almost equally well as PhyloGibbs with phylogeny. PhyME had high sensitivity and outperformed both EMnEM and the non-phylo algorithms at this sensitivity, but it seemed unable to make very specific predictions. EMnEM did not perform better than any of the non-phylo algorithms on these data.

We believe that one important factor contributing to the smaller difference between the phylo and non-phylo algorithms is the limited reliability of the multiple alignments. Since all phylo algorithms only sample configurations consistent with the alignment, any errors in the alignment will hurt their performance. Another factor that probably plays a role is that all phylo algorithms assume that when a site occurs in a conserved block, the site must occur in all species. This is probably not always true, i.e., there are cases where only some of the sequences in an aligned block have retained the site. The non-phylo algorithms can easily deal with this by placing windows only on those sequences that have retained the site, but the phylo algorithms cannot, and a block with several binding sites may be “spoiled” by a single sequence that is missing the site.

All specificities in Figure 6 are significantly lower than those for the synthetic data in Figure 5. There are, of course, many differences between the synthetic and real data (the real data have more complex background, WMs of varying widths, varying numbers of motifs and sites, etc.), but we believe the main reason for the much lower specificity is that the specificities are based on counting only *documented* sites, and that many true binding sites are not yet documented. There is no reason to believe that the algorithms are more likely to recover documented binding sites than they are to recover true but undocumented binding sites. The reported specificities *s_r_* counting only documented sites thus likely underestimate the true specificities *s_t_* by a factor that roughly corresponds to the fraction *f_d_* of all true sites that are documented. That is, assuming that all algorithms are equally likely to recover true but undocumented sites as they are to recover documented sites, and assuming that the algorithm's true specificity *s_t_* matches the specificity *s_p_* that it predicts itself, we have


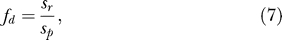


with *s_r_* the reported specificity on documented sites as shown in the left panel of Figure 6. This implied value *f_d_* is shown in the right panel of Figure 6 as a function of the predicted specificity *s_p_*. We see that PhyloGibbs predicted a fraction *f_d_* that was relatively insensitive to *s_p_* and lay between 33% and 45% over a wide range. PhyloGibbs without phylo predicted an *f_d_* in the range of 25% to 40%. Both of these predictions are consistent with the independent estimate [35] that the documented sites represent about 33% of all true binding sites (black line). As with the synthetic data, these results suggest that PhyloGibbs's own assessment of the reliability of its predictions is fairly accurate. Thus, while 18.5% of all predicted sites at a sensitivity of 50% matched documented binding sites, the true specificity is probably somewhere around 55%, and the predicted sites at sensitivity 10% are likely almost all real (A rescaled version of the left panel of Figure 6 is shown in Figure S3). In contrast, the values of *f_d_* obtained for the other algorithms were all unrealistic, for reasons that we already discussed above.

All binding sites that PhyloGibbs predicted in the upstream regions of the genes with one or more sites in SCPD are listed in Dataset S1. They can also be viewed at [36].

### Inferring Yeast's “Regulatory Code”

In the previous sections PhyloGibbs inferred the locations of regulatory sites in one intergenic region at a time. Although sites for a given TF often occur in multiple copies in a single intergenic region, there are also many cases where only a single site occurs, and in those cases PhyloGibbs has to rely on conservation alone to infer the locations of the regulatory sites. However, PhyloGibbs is not limited to run on a single multiple alignment of orthologous intergenic regions but can also run on a set of multiple alignments for co-regulated genes, which should significantly increase sensitivity and specificity.

To test the performance of PhyloGibbs in this setting we used data from a recently published [27] draft transcriptional “regulatory code” of *S. cerevisiae*. Harbison et al. [27] performed ChIP-on-chip experiments with 203 different TFs from *S. cerevisiae* to identify the intergenic regions that are bound by each of them. A suite of six motif-finding algorithms was run on these intergenic regions (several algorithms also used the orthologous regions from other species), and the results were then clustered to arrive at a consensus WM for each TF. When no motif was found computationally for the intergenic regions pulled down, the literature was used, whenever possible, to define a motif. This led to predicted WMs and binding sites for 102 TFs.

We tested PhyloGibbs on the highest confidence set of intergenic regions regulated by each factor. We focused on the 45 TFs that had the fewest binding sites annotated in [27] (a minimum of three and a maximum of 25) since these are generally the most challenging to locate. For 21 of these 45 TFs, the six motif-finding algorithms employed in [27] failed to find a significant motif in the input data, and the reported motif and sites are based entirely on a consensus obtained from the literature (of the remaining 57 WMs with more than 25 or less than three annotated sites, 16 were also solely based on literature.)

We first tested whether, in contrast to the motif-finding algorithms employed in [27], PhyloGibbs was capable of recovering a significant motif that matches the literature consensus for these 21 TFs. For each TF we collected all intergenic regions that were annotated in [27] to contain at least one binding site, collected their orthologs from the other sensu stricto *Saccharomyces* species, produced multiple alignments using Dialign [22], and ran PhyloGibbs on each of these sets of alignments. Since each of these collections of intergenic regions will typically contain binding sites for multiple TFs, we asked PhyloGibbs to search for three motifs, with a total number of sites equaling three times the number of annotated sites for the TF in [27]. The results of this test are shown in Table 1.

**Table 1:**
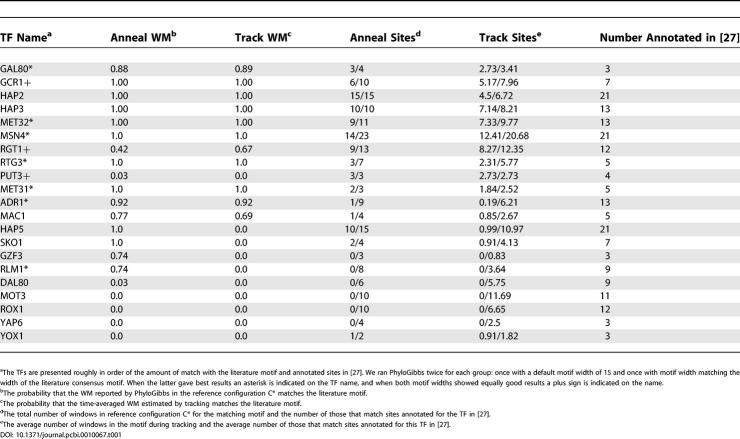
Results of PhyloGibbs on Collections of Intergenic Regions for 21 TFs for Which the Motif-Finding Algorithms in [27] Failed to Recover a Significant Motif but for Which a Literature Consensus Motif Is Available

We evaluated the results that PhyloGibbs reported for each TF in various ways. As described in Materials and Methods, PhyloGibbs reports two WMs for each motif that it finds: one constructed from the configuration C* at the end of anneal, and one by weighing the member windows of a motif with their membership probabilities obtained through tracking. We compared these WMs with the WM that is reported in the literature for each of these 21 TFs. We also compared the sites that PhyloGibbs reported, in both anneal and tracking, with the sites reported in [27]. For each TF we ran PhyloGibbs twice, first with a motif width matching the literature consensus, and then with a default width of 15. We then determined which of the motifs that PhyloGibbs reported best matches the literature motif, and we report a number of statistics for this motif (Table 1). For example, the statistics for TF GCR1 show that, for one of the motifs reported by PhyloGibbs, both the anneal WM and the tracking WM have a probability 1.0 to match the literature WM. There are ten windows with this color in the anneal configuration C*, of which six match sites annotated in [27] for GCR1*.* During tracking, on average 7.96 windows were members of this motif, and on average 5.17 of these members matched sites annotated in [27]. A total of seven sites were annotated for GCR1 in [27].

We see that for 16 of the 21 TFs, PhyloGibbs found a motif that matched, according to at least one statistic, the consensus motif known for this TF in the literature. PhyloGibbs thus apparently outperformed all of the motif-finding algorithms used in [27] on these 16 datasets. For the top eight TFs in Table 1 there is a good match between the WMs that PhyloGibbs reports and the literature WM as well as a significant overlap between the reported sites and the sites reported in [27]. This agreement might suggest that these groups of sites almost exhaustively capture all sites genome-wide for these eight factors. To test this we picked one example, GCR1, and compared the reported sites with known sites from the literature. In [26] there are six genes whose upstream regions have reported GCR1 sites *(TPI1, CDC19, ENO2, ADH1, ENO1,* and *PGK1).* More recently, it was shown that there are additional GCR1 sites upstream of *GKL1* and *GCR1* itself [37,38]. Somewhat surprisingly, of these eight genes only one *(CDC19)* is among the set of four upstream regions containing GCR1 annotated sites in [27]. We ran PhyloGibbs on the eight upstream regions of *TPI1, CDC19, ENO2, ADH1, ENO1, PGK1, GKL1,* and *GCR1,* and recovered a motif that perfectly matched the GCR1 literature consensus. This motif is represented with sites in all upstream regions, although the site upstream of *GKL1* has a posterior probability of only 0.1. Thus, the sites PhyloGibbs found in these upstream regions are very likely true GCR1 sites. This indicates that the sites reported in [27] are far from exhaustive.

The results for PUT3 seem paradoxical. All sites PhyloGibbs reported matched sites reported in [27], but the WMs did not match. The reason for this is that the consensus for PUT3 used in [27], CGG..........CCG, has a 10-bp spacer that is presumed to contain random bases. In contrast, the motif that PhyloGibbs inferred, CGGNNNNGGNTTCCCG, is much more specific. It has been established that *PUT1* and *PUT2* are directly regulated by PUT3 [39]. The upstream region of *PUT2* is indeed among the three upstream regions annotated with sites for PUT3 in [27], but *PUT1* is not. We added this upstream region to the collection of upstream regions for PUT3 and reran PhyloGibbs. PhyloGibbs again found the PUT3 motif, which now included two good binding sites upstream of *PUT1*.

For MET31 the WMs matched reasonably well, and two out of three sites in configuration C* matched, but the sites did not cluster stably during tracking. According to the literature [40–42], MET31 directly regulates *MET25, MET3, MET14, GSH1,* and *MET28*. None of their upstream regions are among the upstream regions annotated with sites for MET31 in [27]. We ran PhyloGibbs on these five upstream regions and found a motif that matched the literature consensus that had sites in all five of these upstream regions that are stable under tracking. Thus, as with GCR1, all these sites are very likely true MET31 sites not annotated in [27].

For ADR1 and MAC1, both reported WMs showed a significant match to the literature motif but the reported sites overlapped only marginally with the sites reported in [27]. For ADR1 there are two genes that have known sites upstream *(ADH2* and *CTA1)* in [26]. Neither of these have annotated sites for ADR1 in [27]. A recent microarray-based study [43] lists 74 genes as under control of ADR1, of which only *PXA1* occurs among the upstream regions with sites for ADR1 in [27]. Both *ADH2* and *CTA1* occur in this list as well. We ran PhyloGibbs on the upstream regions of *ADH2, CTA1,* and *PXA1* and found a motif matching the ADR1 literature consensus and containing sites in all three upstream regions. Again these sites are thus very likely true sites for ADR1 that, except for the *PXA1* sites, are not contained in the annotation of [27]. For MAC1 a similar story applies. Binding sites for MAC1 are listed upstream of *FRE1* and *CTR1* in [26], and *CTR3* and *CTT1* are additionally identified as targets in the literature [44,45]. Of these only *CTR1* is among the genes with sites for MAC1 in [27]. Running PhyloGibbs on these four upstream regions recovered a motif that matched the MAC1 literature consensus perfectly and that had sites upstream of *FRE1* and *CTR1.* It also had a site upstream of orthologs of *CTR3* but, curiously, not in the *S. cerevisiae* upstream region of *CTR3,* which, equally curiously, did not align well with the upstream regions of its orthologs. PhyloGibbs found no site in *CTT1*. This case warrants closer study.

For HAP5 and SKO1, only the anneal WM matched the literature WM. Although a reasonable number of windows occurred on average during tracking for these motifs, there was no stable core. Even the stablest window in each group was only present about 50% of the time. The membership of these groups thus fluctuated significantly during tracking, and this is reflected in the fact that the information score (see Materials and Methods) of the tracking WM is much lower than that of the anneal WM. In [46] five genes *(AHP1, GLR1, GRE2, SFA1* and *YML131W)* are reported as direct targets of SKO1. None of their upstream regions occur among the upstream regions with annotated sites for SKO1 in [27]. We ran PhyloGibbs on the upstream regions of these five genes and found a motif that matches the literature consensus and had sites in the upstream regions of all but *SFA1*. Interestingly, the consensus of the motif PhyloGibbs reported, TTACGTAA, subtly differs from the literature consensus TGACGTCA. The PhyloGibbs consensus is still a palindrome but the second guanine and penultimate cysteine are replaced by thymine and adenine, respectively.

For GZF3 and RLM1 there was only a moderate match of the anneal WM to the literature WM, and no overlap whatsoever of the reported sites with the sites reported in [27]. Coffman et al. [47] report *GAP1, DAL80,* and *UGA4* as direct target genes of GZF3. Again, none of these are among the genes annotated with sites for GZF3 in [27]. We ran PhyloGibbs on the upstream regions of these three genes and recovered a motif that significantly matched the literature motif and had sites upstream of all three genes. Interestingly, the motif that PhyloGibbs reported, GATWAGCGAT, while matching the literature consensus GATAAG, is longer and significantly more specific. Dodou et al. [48] report *RLM1, SMP1, HKR1, KTR2, HSP150,* and *FLO1* as direct targets of RLM1. Of these, only *HSP150* is among the genes with sites annotated in [27]. We ran PhyloGibbs on the set of six upstream regions from [48] and recovered a motif that reasonably matched the *RLM1* literature consensus. The consensus of the motif that PhyloGibbs reported is WGCWAANNTTARAW, whereas the literature consensus is CTAWWWWTAG. PhyloGibbs found sites upstream of four *(SMP1, HSP150, KTR2,* and *HKR1)* of the six genes, with multiple sites in front of *HSP150.*

Finally, there were five TFs (DAL80, MOT3, ROX1, YAP6, and YOX1) for which PhyloGibbs did not find any motif matching the literature motif among the intergenic regions from [27]. DAL80 is one of a family of four GATA TFs that all bind motifs containing the consensus GATA. The experimentally best confirmed target genes for DAL80 are *DAL3, DAL80* itself, *GAT1,* and *UGA4* [47,49,50]. None of these have binding sites for DAL80 annotated in [27]. We ran PhyloGibbs on the upstream regions of these four genes and found a motif with GATAAG consensus that had at least two sites in each of the upstream regions.

Hongay et al. [51] suggest that *ERG2, ERG6,* and *ERG9* are direct targets of MOT3. Again, none of these upstream regions have sites annotated for MOT3 in [27]. We ran PhyloGibbs on the upstream regions of these three genes but did not find a motif clearly matching the literature consensus.

Linde and Steensma [52] found 25 genes in an expression array experiment that are likely targets of ROX1, among which are the three genes *(CYC1, HEM13,* and *ROX1)* with binding sites in [26]. None of these 25 genes are among the genes with ROX1 sites annotated in [27]. We ran PhyloGibbs on the three upstream regions of *CYC1, HEM13,* and *ROX1* and recovered a motif that perfectly matches the literature consensus and had sites in each of the three upstream regions.

For YAP6 a consensus binding site has been established by in vitro studies of different YAP proteins binding DNA [53]. As far as we can tell no clear target genes are known in the literature for YAP6.

Finally, Pramila et al. [54] report 28 target genes for the homeodomain protein YOX1. Of these only *SPO12* is among the genes with sites annotated for YOX1 in [27]. YOX1 is known to interact directly with MCM1, and a motif for YOX1 was reported in [54] that was found by first identifying the MCM1 binding sites in the upstream regions of the target genes, and then searching for an additional overrepresented motif near the MCM1 sites. We also ran PhyloGibbs on the upstream regions of these 28 genes and identified a motif with consensus ATTACWTTTCCYNAW. The right end of this consensus matches the *MCM1* consensus tttCC.rAt..gg, and the left end corresponds to the standard homeodomain core ATTA. This motif has sites in about half of the 28 upstream regions. Note that Pramila et al. [54] report a YOX1 consensus of yaATTa that differs from the consensus, AsAATA.TGAmr, that is reported in [27].

Table 2 shows the results of running PhyloGibbs on the remaining 24 TFs with fewer than 25 sites in [27], where their computational methods did produce a motif. The table shows that for 18 of these, both the anneal and tracking WM from PhyloGibbs matched the WM in [27], along with a significant fraction of the sites. As with the case of GCR1 above, one should not conclude from this that the reported sites in any way cover all the true sites for these TFs. For four TFs, a motif that PhyloGibbs reported had some overlap with the motif reported in [27], and there was no meaningful overlap for only two cases: SPT2 and SPT23. We examined in detail only these two cases.

**Table 2:**
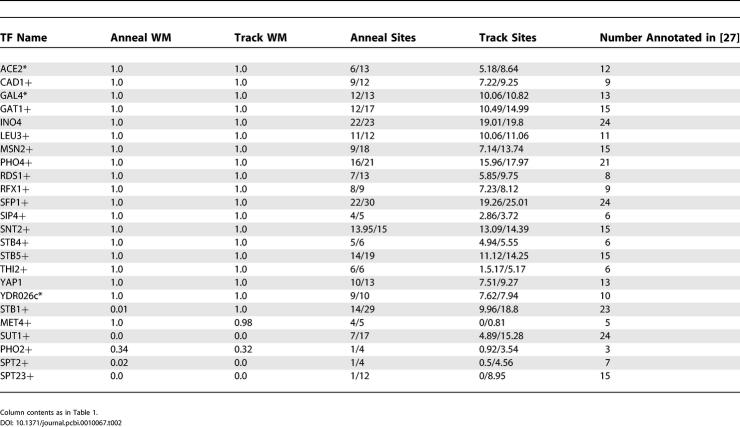
Results of PhyloGibbs on Collections of Intergenic Regions for 24 TFs for Which the Motif-Finding Algorithms in [27] Found a Significant Motif

The protein SPT2 is involved in regulation of chromatin structure and is known to interact directly with the SWI/SNF complex and with histones. SPT2 has been reported to not have any sequence specificity in its DNA binding [55], and more recently Novoseler et al. [56] have proposed that SPT2 binds to two strands of double-stranded DNA at their crossing point. Moreover, the only well-established target of SPT2 that we found in our cursory survey of the literature was the *HO* gene, and this gene is not among the genes annotated with sites for SPT2 in [27]. We thus believe that the motif reported in [27] is dubious. It is well established that SPT23 regulates *OLE1* expression [57], but this gene is not among the genes with sites for SPT23 annotated in [27]. Given that SPT23 does not seem to have a DNA-binding domain, it is likely that SPT23 functions as a cofactor and lacks specific DNA binding on its own.

In summary, PhyloGibbs, when run on the highest quality intergenic regions and their orthologs reported in [27], found a motif that matches the literature consensus for 16 of 21 TFs, where the computational methods of [27] failed. For 11 TFs (ADR1, DAL80, GCR1, GZF3, MAC1, MET31, MOT3, RLM1, ROX1, SKO1, and YOX1), where the correspondence was weakest or nonexistent, we extracted co-regulated groups of genes from the literature. In every case there was little or no overlap between the literature list and the set of regulatory targets claimed in [27]. For all but one of the 11 (MOT3), PhyloGibbs found a motif that matched the literature consensus and reported sites in all or almost all of the upstream regions. Thus, when a solid motif was not found, the problem was likely with the set of intergenic regions in [27], not with PhyloGibbs.

Detailed comparisons of PhyloGibbs's results with the annotations of [27] are in Dataset S2. A list of all binding sites predicted by PhyloGibbs for the 45 TFs is in Dataset S3. They can also be browsed at [36].

## Discussion

Motif discovery algorithms make use of a variety of different kinds of information to identify binding sites for regulatory factors in intergenic DNA. Sequence specificities for particular regulatory factors can sometimes be obtained through detailed experimentation, including DNaseI footprinting and SELEX experiments. Weight matrices representing the sequence specificities can then be used to locate putative binding sites for these regulatory factors. In this respect algorithms often look for combinations of binding sites for several WMs [58–60] that occur within a relatively short interval on the genome. Ab initio methods typically operate on sets of sequences that are thought to contain binding sites for common regulatory factors. To isolate such sets of sequences various kinds of experimental data, such as data from microarray or ChIP-on-chip studies, can be used. The algorithms then search for sequence motifs that are overrepresented among the sequences. Alternatively, ab initio methods can use orthologous sequences from related species to search for short sequence segments that appear more conserved evolutionarily than surrounding sequences, or more conserved than can be expected based on the evolutionary distances of the species.

In this paper we have presented a novel algorithm for ab initio discovery of regulatory sites that combines the search for overrepresented motifs with the analysis of sequence conservation in arbitrary collections of sequences and their orthologs. A major challenge in using orthologous sequences is distinguishing conservation due to functional constraints, such as regulatory sites, from conservation simply due to evolutionary proximity. In order to do this correctly one has to determine which sequence segments have evolved from a common ancestral segment, i.e., the sequences have to be aligned, and their phylogenetic relationships have to be taken into account. This is complicated by the fact that orthologous intergenic sequences typically cannot be trivially aligned but show a complex pattern of conserved blocks interspersed with unalignable segments. Moreover, regulatory sites are not necessarily restricted to the conserved blocks.

Focusing only on the conserved blocks, as is done in phylogenetic footprinting approaches [13–17], misses a significant fraction of true regulatory sites, and we thus chose to include all sequence. Ideally, one would sample over all possible combinations of multiple alignments and binding site configurations, but we believe that this search space is too large to search effectively. Moreover, especially for relatively closely related species, large sequence blocks can be unambiguously aligned and the search space can be significantly reduced by pre-aligning these. However, pre-aligning all orthologous sequence groups in global multiple alignments could be deleterious because global alignment often “forces” phylogenetically unrelated sequence segments together in the alignment and might introduce spurious gaps into binding sites. We thus prefer to align only those sequence segments that can be unambiguously aligned and leave the rest of the sequences unaligned. In our current implementation we use the Dialign [22] algorithm to create multiple alignments. It searches all pairs of statistically significant (over some cutoff) pairwise ungapped alignments and then, starting from the most significant, combines all mutually consistent ones into a multiple alignment. The parts of the sequences that are not part of the consistent set of pairwise alignments are left unaligned.

Recently, two algorithms [24,25] were reported that generalize the EM algorithm MEME [6] to include phylogeny. These algorithms use EM to search the space of WMs as opposed to sampling the space of binding site configurations as PhyloGibbs does. One important advantage of the latter approach is that any arbitrarily complex prior *P*(*C*) can be easily implemented, whereas the EM approaches are essentially restricted to putting an independent prior probability on each binding site occurrence. As a consequence, we have observed that the number of sites that PhyME and EMnEM predict increases or decreases dramatically as the branch lengths of the phylogenetic tree are changed, whereas PhyloGibbs's predictions are much less sensitive to the phylogeny parameters.

Another difference, also related to the prior *P*(*C*) over configurations, is in the way that multiple motifs are treated. The EM algorithms search for multiple motifs by searching for a single motif at a time, blocking its sites and iterating. In contrast, we have optimized PhyloGibbs's move set such that it can efficiently search for sites for multiple motifs in parallel. This also allows us, as we intend to do in the future, to extend PhyloGibbs's scoring function to take correlations between the positions of binding sites for different motifs into account. Finally, as shown and discussed in the results on synthetic and real data, only PhyloGibbs realistically estimates the reliability of the binding sites that it reports. The EM algorithms ignore the uncertainty associated with the WM they infer and therefore vastly overestimate the significance of the sites that they predict.

An important novel feature of our algorithm is the anneal-and-track strategy. The algorithm first uses simulated annealing to search for the configuration C* with maximal posterior probability *P*(*C*|*S*) and uses this as the reference configuration. In the second phase it then samples the distribution *P*(*C*|*S*) of all binding site configurations and compares these samples with the reference configuration C* to assign posterior probabilities to all sites it reports. This strategy allows the algorithm to assign realistic posterior probabilities to all the sites that it reports. Instead of using the annealing step, users can also specify a reference configuration C* themselves and use the algorithm to assign posterior probabilities to the motifs occurring in C* and the sites associated with them. The anneal-and-track strategy also makes the algorithm robust to prior overestimates of the number of motifs and sites that occur in the data. Superfluous sites found by the anneal will not be stably associated with a color during tracking, and superfluous motifs will have minimal membership during the tracking.

In some approaches multiple runs of one or more algorithms on the same data are used to assess motif significance. However, in order to assess which motifs recur in multiple runs, results from the different runs have to be clustered, and the only way to do this correctly is to use the same sampling method as was used to extract the motifs in the first place. Our tracking strategy circumvents the need for such post-processing of the results.

Our tests with synthetic data showed that, in the idealized situation where orthologous sequences are perfectly aligned, algorithms that take phylogeny into account significantly outperform those that do not (see Figures 3 and 5). We also showed that, given enough species and a reliable alignment, even a single site for a fuzzy motif can be accurately recovered (see Figure 4). This underscores the potential power of using orthologous sequences for regulatory site detection. It suggests that any regulatory site can be reliably recovered given an alignment of enough related species in which the given regulatory site occurs.

We used intergenic regions of *S. cerevisiae* that contain experimentally verified [26] binding sites, together with those of four other sensu stricto *Saccharomyces* species to test the extent to which different algorithms can recover regulatory sites from multiple alignments of single intergenic regions. We ran PhyloGibbs and four other motif-finding algorithms on the multiple alignments of 200 intergenic regions and showed that PhyloGibbs outperforms all other algorithms including EMnEM and PhyME, which also take phylogeny into account (see Figure 6).

We also ran PhyloGibbs on collections of intergenic region alignments of genes that were annotated in [27] to contain binding sites for a common TF based on data from ChIP-on-chip experiments. For almost all cases for which the motif-finding methods in [27] found a significant motif, PhyloGibbs reports a matching motif. More importantly, for 16 of the 21 TFs for which the six motif-finding methods in [27] failed to find a significant motif, PhyloGibbs does report a motif that matches the literature consensus. We studied in detail those TFs for which there was disagreement between PhyloGibbs's results and those in [27]. For all these TFs we found that the gene sets reported in [27] have very little overlap with targets reported in the literature. Moreover, when PhyloGibbs is run on the upstream regions of the literature targets it recovers a set of binding sites that match the literature in all but one case.

There are several issues that we intend to address in future extensions of the algorithm. First of all, we intend to extend the types and specificity of the priors that we allow. For example, when running on multiple alignments of several different upstream regions, one may sometimes have prior information that *each* upstream region has at least one site for a particular TF. We would thus like to allow for priors that specify not just the total number of sites, but specifically the likely number of sites in each upstream region. We also intend to extend the priors on WMs. In many cases one may already possess alignments of known sites or other specific prior information on the sequence specificity of particular WMs, and we will extend the algorithm to allow users to “seed” different colors with such specific prior information. These extensions are all straightforward to implement. A more challenging issue for the future is the improved treatment of the multiple alignment of the intergenic regions. The complex patterns of conserved blocks interspersed with unalignable sequence that is observed in multiple alignments of orthologous sequences suggests that the evolution of intergenic regions is currently not well understood. Different mechanisms that lead to insertions and deletions of various sizes, such as tandem duplications [61], probably play a significant role, and current alignment algorithms do not take such events into account. Another important facet that is currently mostly unexplored is the extent to which regulatory sites are conserved across related species. Intuitively one expects that the closer the species, the more binding sites will be shared between them, but it is currently not generally known what fraction of sites turns over as a function of evolutionary distance, and how much this varies with the TF and evolutionary lineage in question. Finally, it is clear that different binding sites have different affinities for their cognate TF, and it is conceivable that binding sites are selected in evolution not only to remain recognized by their TF, but that there is specific selection for preserving the strength of the binding site. Further investigation will be necessary to determine if realistic models of binding site evolution need to take such selection for binding site affinity into account.

## Materials and Methods

### WM information score.

The most useful quantity characterizing the “quality” of a WM *w* is its information score *I:*


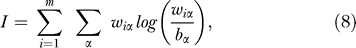


where *m* is the width of the WM, *b*_α_ is the background probability of base α, and the logarithm is often calculated base 2 to express the information score in bits. Many relevant quantities regarding sets of binding sites can be expressed in terms of information scores. For instance, the fraction of random sequences of length *m* that “match” the WM scales as *e^−I^*. The probability that a sample of *n* random sequences will have base counts *n_i_*_*α*_ = *nw_i_*_*α*_ is approximately *e^−nI^*. Similarly, the likelihood ratio *R* of this sample of sequences stemming from a WM versus stemming from background scales as *R* ∝ *e^nI^*. The information score thus accurately summarizes the sequence specificity of the set of binding sites that it represents.

### Prior on configurations.

The simplest prior over configurations, representing “complete ignorance,” is the uniform prior, *P*(*C*) = constant, that assigns equal probability to all configurations. However this prior is “too ignorant” to work well in practice. In particular, the large majority of all configurations will consist of configurations with a very large number of windows that essentially cover the entire input data. PhyloGibbs thus allows for two kinds of priors that take into account information regarding the expected total number of sites and motifs in the dataset. First, one can run PhyloGibbs on only the subspace of configurations with a fixed number of colors *c* and a fixed number of total windows *N* (effectively setting *P*(*C*) = 0 for all configurations outside of this subspace). The second way of putting a prior on the space of configurations is by introducing an exponential prior distribution over the number of colored windows:


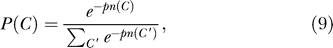


with *n*(*C*) indicating the number of colored windows in configuration *C*. For each value of *p,* the distribution *P*(*C*) is the maximum entropy distribution over configurations conditioned on the expected number of colored windows 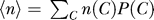 . One can thus use this prior to incorporate prior knowledge of the expected total number of binding sites in the input data.

### Derivation of *P*(*S_c_*).

The probability *P*(*S_c_*|*w*) that all sequences *S_c_* in the windows belonging to color *c* were drawn from a particular WM *w* is given by


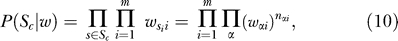


with *m* being the width of the WM and *n*_*αi*_ being the number of times that base α occurs at position *i* among the sequences in *S_c_*. Since we do not know the WM, we integrate over all possible WMs (separately for each color) [28]. That is, we formally have


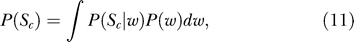


where *P*(*w*) is a prior distribution over the space of WMs, and the integral extends, for each position *i,* over the simplex *w*_α*i*_ ≥ 0 and 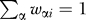 . PhyloGibbs uses Dirichlet prior distributions of the form


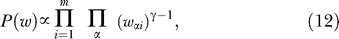


where the γ parameter, which is generally referred to as a pseudocount, can be set by the user (default is γ = 1). With this prior the integral can be done exactly, and we obtain


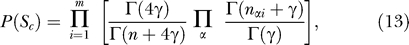


with *n* being the total number of sequences in color *c* and Γ(*x*) the gamma function.

### Background model.

We will assume that the background sequence was generated by a Markov model of order *k,* where *k* is specified by the user and may run from zero to arbitrary length (within reason). In this model, the probability of observing a background base α*_i_* depends on the *k* preceding bases α*_i − k_* through α*_i −_*_1_. We estimate the probabilities *P*(α*_i_*|α*_i −_*_1_…α*_i − k_*) either from a large set of intergenic sequences provided by the user or from the set of sequences that are being sampled as follows:


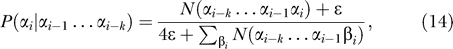


where *N*(α*_i − k_*…α*_i_*) is the actual number of occurrences of the string α*_i − k_*…α*_i_* in either the large intergenic sequence or the input data. The pseudocount ɛ can again be set by the user. Using this model, the probability for all the uncolored sequences is


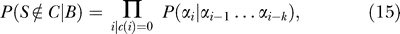


where the product is over all positions *i* that have color zero.

It is conceptually and computationally convenient to divide the probabilities *P*(*S*|*C*) by the probability *P*(*S*|*C*_0_) of the configuration *C*_0_ in which all windows are color zero (i.e., background). The factor *P*(*S* ∉ *C*|*B*) then cancels, and we have


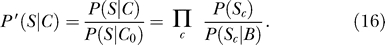


For each color *c* the denominator can be calculated in the same way as the numerator, with background probabilities replacing the WM entries *w*_α*i*_ *,* and with no integral.

### Identifying legitimate windows.

At the start of each run, PhyloGibbs determines the set of all legitimate windows in the data. That is, it finds all locations where a window of length *m* can be placed, extends the window to contain sequences from all species that share aligned bases in this segment, checks for consistency of the alignment, completes the window with unaligned bases when necessary, and records all other windows that overlap this window. Formally, the procedure is as follows. All bases in all sequences are first set as “unmarked.” All bases are then examined sequence by sequence from left to right. If the current base is “ummarked,” a single-sequence window of width *m* is constructed starting at that base. Next, for every uppercase letter in the window all other sequences that contain an uppercase letter in that position are added until no more sequences can be added. (The expanded window will now contain, for every uppercase letter in it, all sequences where an uppercase letter occurs in that position.) The window is then examined for consistency: the relative positions of vertically aligned uppercase letters should be the same for all uppercase letters (i.e., there should be no gaps, or if gaps exist, they should extend across all sequences affected by the vertically aligned letters). A consistent window is accepted. The treatment of inconsistent windows depends on the settings of the −D option. If −D 2 is used, all inconsistent windows are simply rejected. If the −D 1 option is used, the inconsistent window is split into smaller consistent windows as follows. Recursively, the first sequence in the current window is picked and all other sequences consistent with it are collected. This process is then repeated on the remaining sequences until all sequences have been used. It is possible that the choice of splitting is dependent on the order of sequences (e.g., when three sequences are mutually inconsistent but separating any one of them renders the others consistent). Currently we ignore this complication and assume that it is uncommon in practice. Finally, the window or windows thus obtained are added to the list of available windows, and the first base for each sequence in a window is marked so that the program will not attempt to construct additional windows starting at those positions.

### Derivation of *P*(*W*|*w*) under the evolutionary model.

Our model for the evolution of binding sites assumes that all bases mutate at a constant rate γ. When a base at position *i* of a binding site is mutated to letter α, we assume that the probability that selection will fix this mutation is given by the WM component *w*_α*i*_. Under this simple model, the probability that a base at position *i* will mutate from β to α over a time *t* is given by [60]


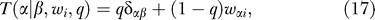


where we have introduced the “proximity” *q,* which corresponds to the probability *q* = *e* ^−γ*t*^ that no mutation took place at this position during time *t*.

Note that as *q* → 0 the expression reduces to the probability *w*_α*i*_ of observing an independent base α at position *i* when sampling from the WM *w*. Also note that the expression has the correct composition property when an intermediate ancestor *a* is inserted:


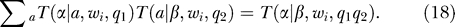


To calculate the probability *P*(*W_i_*|*w_i_*) of observing the set of bases *W_i_* in column *i* of a window *W,* we next need the phylogenetic tree relating the sequences in the alignment. The phylogenetic tree of the yeast species that we used in our runs on real data is well approximated by a “star” topology, and the calculation of *P*(*W_i_*|*w_i_*) simplifies significantly for this case. We thus first present the derivation for star topologies and indicate below how PhyloGibbs calculates *P*(*W_i_*|*w_i_*) for more general topologies.

For a star topology, the probability *P*(*W_i_*|*w_i_*) of obtaining the set of bases *W_i_* at column *i* of the window *W* given the WM column *w_i_* is given by


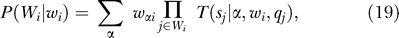


where *j* runs over all the sequences in the window, *s_j_* is the base appearing at position *i* in sequence *j,* and *q_j_* is the proximity of sequence *j* to the ancestor. Since the ancestor sequence is assumed to be a sample from the WM, we assign a probability *w*_α*i*_ to the possibility that the ancestor had base α at position *i,* and sum over all possibilities *α*. For the whole window we of course have


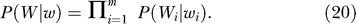


The expression *P*(*W_i_*|*w_i_*) is a polynomial in the WM components *w*_*αi*_ *,* which, as can be shown with a little algebra, has *N* + 4 monomial terms of the form 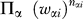 for an alignment of *N* sequences. As in the previous section, we will need to integrate products of these polynomials for multiple windows, i.e., we need to calculate integrals of the form∫*P*(*W_i_*|*w_i_*) *P*(*W′_i_*|*w_i_*)*P*(*W′′_i_*|*w_i_*)…*dw_i_*. While these integrals can be done analytically, the number of terms involved equals (*N* + 4)(*N′* + 4)(*N′′* + 4)…, and this quickly becomes unwieldy when the number of windows grows. Therefore, in practice we approximate the single-window polynomials *P*(*W_i_*|*w_i_*) with monomials.

### Approximation of WM integrals.

For each aligned window, we approximate the window column function *P*(*W_i_*|*w_i_*) of equation 19 by a monomial of the form


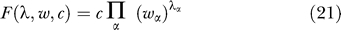


(in this section we drop the position index *i* for notational simplicity). This form has the advantage that the integral over *w* for the product of an arbitrary number of functions of this form is straightforward:


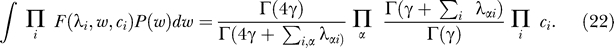


Therefore, using this form we can easily calculate the integrals for an arbitrary number of windows.

We now choose to set the parameters *c* and λ_*α*_ such that the first moments of the distribution *P*(*W*|*w*) and *F*(λ,*w,c*) match. For the zeroth moment (normalization) this gives


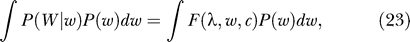


where *P*(*w*) is a prior over the WM space. For the first moment we demand


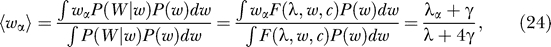


for all *α*, where 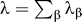 and γ is the pseudocount of the prior 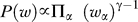 . Note that the first and last equality in equation 24 are always true and that our demand is the middle equality. These equations allow us to fix *c* and the ratios λ_*α*_/λ but leave the overall scale λ still free. We use λ to approximate the second moments. We thus demand that the exact second moments


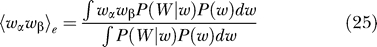


match the second moments of the approximation


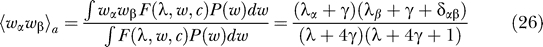


as “closely” as possible. This could, for instance, be done by choosing λ such that the square-deviation is minimized. In the current implementation we set λ by, for every combination of α and β, solving for λ from the equation 〈*w*_α_ *w*_β_〉*_e_* = 〈*w*_α_ *w*_β_〉*_a_*. This yields


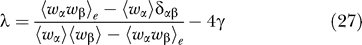


We then set λ equal to the average of the λs that are obtained from this equation for the 16 combinations of α and β.

In calculating the parameters λ_α_ and *c,* all the complicated exact polynomial integrals for the single windows need to be done. However, since we only need to do this once for every window at the start of the run, and store the results, this does not incur a significant computational cost.

Finally, it is clear that by demanding that we approximate the function *P*(*W*|*w*) by a monomial of the form 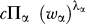 we are making an uncontrolled approximation. In addition, we set the λ_α_ and *c* by fitting the zeroth and first, and approximating the second, moments of the distribution *P*(*W*|*w*), and it is not clear that this is the “best” choice one can make. For instance, one could imagine fitting the λ_α_ and *c* such that the average square-deviation of a much larger number of moments (including moments of high order) is minimized. However, numerical experiments for a number of windows with different parameters show that, even for fairly high-order moments, in almost all cases our current approximation is quite accurate (see Table S1).

### General phylogenies.

The above method of treating a star-topology phylogeny can be readily extended to deal with more general situations. A completely general phylogeny (assuming no “lateral transfer” of DNA) can be represented as a tree; the root is the last common ancestor of all given species, nodes are intermediate ancestors (last common ancestor of some, but not all, given species), and the leaves are the actual species under consideration. All unknown ancestors (root and nodes) are separately summed over. Proximities *q* measure the distance of leaves or nodes to the previous node, not necessarily the last common ancestor.

Consider such a phylogenetic tree that does not have a star topology (i.e., contains internal nodes other than the root). At least one of the intermediate nodes must be such that all its children are leaves. Let the unknown base for this node at column *i* be β (summed over), and let the base for its parent node (again, not necessarily the root) be α. The subtree below β contributes a factor *P*_β_ to the total probability, given by


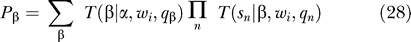


where the full expression would contain other factors involving α as well as a sum over α; *q*_β_ is the proximity of β to its immediate ancestor α, the product runs over children of β indexed by *n, s_n_* is the base of the *n*th descendant, and *q_n_* is the proximity of the *n*th descendant to β. *T* is given by equation 17. In particular,


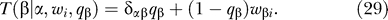


Substituting this into equation 28, we get two terms:


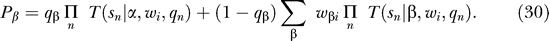


The first term simply removes the node β and attaches all its children to α (with unchanged proximities). The second term—identical, apart from a prefactor, to equation 19—can be treated as an independent factor to anything it multiplies, completely decoupled from the sums over α and other ancestors. In other words, with probability *q*_β_, base β is the same as α and all its leaves can be attached directly to α, and with probability 1 − *q*_β_, base β is mutated from α and can be treated as a new, independent ancestor for all of its descendants, disconnected from the rest of the tree.

By repeating this process, one can reduce any tree to a sum of products of star-phylogeny subtrees with appropriate prefactors. PhyloGibbs then applies the monomial approximation described in the previous section to each of the star-phylogeny subtrees, as well as to the final sum. Note, however, that the number of terms involved may grow exponentially with the number of species. As the number of species becomes large we thus need to make additional approximations to make this procedure computationally feasible.

### Move set.

A single time step of the algorithm consists of a “cycle” of a fixed number of moves of each of the types outlined in the following paragraphs.

Window-shift moves preserve the total number of colors, and the total number of colored windows (but may redistribute the windows among existing colors). We choose one of the presently colored windows at random. If it is the only one in its color, we make no operation (but to ensure detailed balance we update the time counter by one). If it is not the only window in its color, we color it zero (i.e., deselect it), and choose a new window from all of the available color-zero windows (including the window we selected) to replace it. The new window can have any of the existing colors, not necessarily the same as the window it is replacing. This move is computationally expensive, since if there are *N* available windows and *c* available colors, we have to calculate the scores for *Nc* potential moves, but it allows for rapid convergence.

Color-change moves allow for changes in the number of windows and the number of colors, while satisfying detailed balance. We select any of the existing windows, including color-zero windows. If the chosen window overlaps a non-zero-colored window then this window is blocked and we make no operation (but update the time counter). Otherwise, we reassign a color to the window, which may be zero, one of the existing colors, or a new color. Note that if the window was the only one in its color, a “new color” means “the same color as before.” The window-shift moves are not ergodic by themselves because they stay inside a subspace of fixed *N* and *c* (respectively, number of colored windows and number of colors). The color-change moves are ergodic but do not have good convergence properties, so an alternation with window-shift moves is desirable.

With the previous two moves it is possible for the sampler to get stuck in a local optimum where the windows in a given color are all shifted by an equal amount from their best location. The global shift move addresses this problem. This move picks a color at random, and samples all ways of coherently shifting every window in that color by a fixed amount without “colliding” with an already-colored window.

Maskbit-flip moves are the final move type. Long motifs tend to be fuzzy, and not every position is sharply defined. Sometimes, the score of a collection of sites can be improved by scoring a subset of its columns according to the background model rather than assuming they derive from a WM. We thus allow the “masking” of certain columns, comparing whether or not the overall score is improved by scoring them according to background. For each color we maintain a mask, and sample over the states (zero or one) of the mask bits. In our experience, allowing such masking can increase performance for long motifs that contain nonconstrained sequence, such as occur in bacteria when TFs bind as dimers. However, for short motifs the enlargement of the configuration space that is associated with these masks may result in poorer discrimination.

### Tracking.

After each cycle during the tracking phase, the best-matching color *c̃* from the current configuration *C* is found for each color *c* of the reference configuration C*. To do this we define a match *M*(*c,c̃*) between colors *c* and *c̃* as follows. For all shifts −*m*/2 ≤ *s* ≤ *m*/2 (the window width is *m*), we shift all the plus-strand windows in *c̃* by *s* to the right and all the minus-strand windows in *c̃* by *s* to the left. Note that, since different parts of the multiple alignments will span different subsets of species, we have to define carefully what we mean by the word “shift.” We only consider two windows *X* and *Y* shifted versions of each other if they both span the same set of species, and if the position of the start of the window is shifted by the same amount in each of the sequences in the windows. Thus, in general, shifted versions will only exist for some of the windows in *c̃*.

After shifting all the windows in *c̃* by an amount *s,* we then count the number of shifted windows *n*(*s*) that exactly match a window in *c*. The score *M*(*c,c̃*) is given by


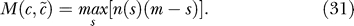


Note that this corresponds to the maximal amount of overlap between the sites in *c* and the sites in *c̃* when counting only sites in *c̃* that are shifted by a common amount with respect to *c*. We also keep track of the shift *s* at which the maximum occurred. Once we have determined the color *c̃* that maximizes *M*(*c,c̃*) and the shift *s* at which the maximum occurred, we then add a count of one to *n*(*w,c*) for every window *w* that is obtained by shifting the windows in *c̃* by *s*. The counts *n*(*w,c*) record the number of times that each window *w* is associated with reference color *c*. At the end of tracking we divide the counts *n*(*w,c*) by the total number of cycles *n* to obtain *p*(*w,c*) = *n*(*w,c*)/*n*. For each reference color *c* PhyloGibbs reports all windows *w* for which *p*(*w,c*) ≥ *p*_min_ sorted from large to small *p*(*w,c*). These lists of membership probabilities provide a summary of the distribution *P*(*C*|*D*) using C* as a reference configuration.

### Synthetic data runs.

For Figure 3 we generated random intergenic ancestral sequences of length *L* = 500 with *s* = 4 sites sampled from a single WM. For the different panels in Figure 3 we used WMs of width *m* = 10 with polarizations *p* = 0.6, *p* = 0.75, and *p* = 0.9 and random WMs for the lower right panel. For the data in each panel we generated gapless alignments of *S* = 5 descendant sequences at proximities *q* = 0.1 through *q* = 0.9 in steps of 0.1. For each parameter setting we generated 50 different synthetic datasets. PhyloGibbs with phylogeny was asked to search for four multi-sequence windows for a single WM of width ten, to assume the correct proximity *q* for all species, and to use a background model where each base occurs with probability 1/4. The non-phylo algorithms treat the five input sequences as independent and were asked to search for 20 single-sequence sites for a WM of width ten. The precise command lines employed were as follows: for PhyloGibbs with phylogeny, –D 1 –G *q* –m 10 –I 4 –f inputfile –N −1; for PhyloGibbs without phylogeny, –D 0 –m 10 –I 20 –f inputfile –N −1; for WGibbs, –PBernoulli inputfile 10 20 –Z –n; and for MEME, inputfile –dna –mod anr –w 10 –nsites 20 –nmotifs 1 –nomatrim –revcomp –maxiter 500 –text –nostatus.

As a measure of performance we took the fraction of all the bases in real sites that overlapped predicted sites and averaged it over all datasets for each parameter setting. This “overlap” thus runs from zero to one. For each parameter setting the standard error of the overlap is given by


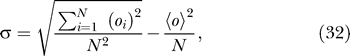


where *N* is the number of datasets, *o_i_* is the overlap for dataset *i,* and 〈*o*〉 is the average overlap.

For Figure 4 we generated random ancestor sequences of length *L* = 500, embedded a single site for a random WM of width ten, and created *S* descendant sequences of proximity *q* = 0.5 for *S* running from two to 30. For each value of *S* we created 250 datasets and ran PhyloGibbs with the following command line settings: –D 1 –G 0.5 –m 10 –I 1 –N −1 –f inputfile. For each *S* we calculated the average overlap 〈*o*〉 and standard error as in equation 32.

The data for Figure 5 were generated, for each of 250 intergenic regions, by picking three random WMs of width *m* = 10, sampling three sites from each, embedding them in a random ancestral sequence of length 750, and creating five descendant sequences at *q* = 0.5. The phylo algorithms were asked to find sites for three WMs of width ten, with an expected number of three (multi-species) sites per WM. They were all given the correct phylogenetic tree (with star topology) and (gapless) multiple alignment. The non-phylo algorithms treat the five descendant sequences from each synthetic intergenic region as independent and so the expected number of sites per motif was set to 15 for these. The command-lines employed were as follows: for PhyloGibbs with phylogeny, –D 1 –G 0.5 –m 10 –I 3,3,3 –N −1 –f inputfile; for PhyME, –N 1 infile blkfile –w 10 –nmotifs 3 –revcompW –ot 0.05 –nsites 3 –niter 50 –nseediter 10 –K 5 –pf phylogenytree.txt –tree; for PhyloGibbs without phylogeny, –D 0 –m 10 –I 15,15,15 –N −1 –f inputfile; for WGibbs, –PBernoulli inputfile 10,10,10 15,15,15 –F –Z –n –i 1500 –S 30 –p 60; and for MEME, –dna –mod anr –w 10 –nsites 15 –nmotifs 3 –revcomp –nomatrim –maxiter 500 –nostatus. EMnEM uses a control file. The relevant parameters that we set were as follows: –w 10 –e 3.0 –b 1 –n 3 –u 1 –r 0 –m 0.

To produce the left panel of Figure 5 we recovered, for each algorithm, all the predicted sites and sorted them by their posterior probability. We then obtained a series of sublists *l_i_* by excluding all predicted sites below a cutoff posterior probability *c_i_*. We chose the cutoffs *c_i_* such that at *c*_0_ all predicted sites were included in *l*_0_, at *c*_1_ all but the last 100 sites were included in *l*_1_, and generally *l_i_* had all but the bottom 100*i* predicted sites. For each list *l_i_* we then calculated the number of bases *A* in all intergenic regions that were hit by sites in this list, the total number of bases *T* in true (planted) sites, and the number of bases *I* in the intersection of these two sets. Given these counts, the sensitivity is given by *I/T* and the specificity is given by


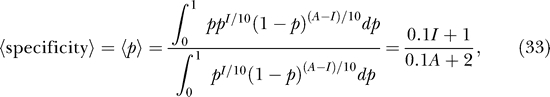


and the standard error we similarly estimate as


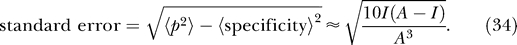


This estimate of standard error correctly takes into account the fact that as the number of predictions *A* gets larger, our estimate of the algorithm's specificity becomes more precise. The factors ten and 0.1 are there to (approximately) take into account that the *A* predicted bases are not all mutually independent but that they come in windows of *m* = 10 consecutive bases.

For the right panel of Figure 5 we used the same estimates of the specificities and their standard errors, but plotted these as a function of the specificity sp*_i_* that the algorithm predicts for each sublist *l_i_*. This predicted specificity sp*_i_* for the list *l_i_* is obtained by averaging the posterior probabilities of all the predicted sites in list *l_i_*.

### Yeast data runs.

We “cleaned up” the dataset of experimentally documented binding sites from the SCPD [26] as follows. In its original form it contained 726 binding sites regulating 234 different genes. We removed sites that either lay in coding regions or that lay more than 1,000 bp upstream from translation start, and fused the overlapping sites that remained. After this we were left with 466 sites for 200 genes.

The upstream regions of the 200 *S. cerevisiae* genes and their orthologs were obtained from the *Saccharomyces* Genome Database [62]. For the sequence of *S. paradoxus* we used data from the MIT group [16], while for *S. bayanus, S. mikatae,* and *S. kudriavzevii* we used data from the Washington University group [15]. For each group of orthologs we took either the entire intergenic region up to the neighboring coding sequences, or 1,000 bp, whichever was shorter. Not all genes in our set of 200 have orthologs assigned in all other species. There were a total of 796 intergenic regions from five species for our 200 genes. This means that on average we had four orthologs per gene. We did not cross-check the accuracy of the annotation of orthologous genes in the downloaded files. Instead, we performed the following simple test: we flagged a 5′-UTR sequence as “dubious” if it had fewer than 100 bases or if, after aligning with Dialign, fewer than one in ten bases were marked as “aligned” (i.e., in capitals) with other sequences. Only 20 out of these 796 sequences got flagged by one of these criteria; we therefore believe that over 97% of the orthologous sequences are likely accurate, and we simply retained all in our runs.

We aligned each set of orthologous intergenic regions with Dialign [22] using the following command line: dialign2–2 –n –thr 5 –fa infile, where the setting –thr 5 ensures that only significant blocks will be aligned. We discovered, however, that the current implementation of Dialign (version 2.2.1) has a severe bug in the way it formats and displays its output. Unrelated sequence segments are sometimes reported in such a way that it appears they are aligned. For example, if one feeds Dialign four sequences, two of which contain all adenines, and two all cysteines, then Dialign will appear to align all of these together without gaps, as opposed to two blocks of pairs (the authors have been informed privately). The bug is in the assembly of fragments to the output file, and we used a wrapper, written by Michael Mwangi, to correctly assemble the fragments. Meanwhile we developed our own multiple-alignment algorithm, Sigma, which was not yet used for the results reported in this paper and which will be described elsewhere (R. Siddharthan, unpublished data).

For PhyloGibbs, EMnEM, and PhyME we needed to specify the phylogeny of the *Saccharomyces* species. The topology of the species tree can be determined unambiguously [63]. It has *S. cerevisiae* on one end and the other species branching off from it in the following order (from near to far): *S. paradoxus, S. mikatae, S. kudriavzevii,* and *S. bayanus*. We approximated this tree by a star topology. For PhyloGibbs we needed, for each of the species *i,* the probability *q_i_* that since the common ancestor no mutation took place in any given base. These “no mutation” probabilities, which we call “proximities,” can be best estimated by looking at the conservation statistics of “neutral” positions in the genome. It was recently shown [35] that conservation rates between *S. cerevisiae* and the other species at third positions of 4-fold degenerate codons are approximately constant across the genome. The conservation rates *c* reported in [35] are *c*_cer,par_ = 0.74, *c*_cer,mik_ = 0.6, and *c*_cer,bay_ = 0.52. In the approximation of a star topology, the conservation rates *c_i,j_* are given in terms of the proximities *q_i_* and *q_j_* through


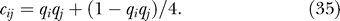


Assuming that *q*_cer_ = *q*_par_ we obtain *q*_cer_ = *q*_par_ = 0.8, *q*_mik_ = 0.58, and *q*_bay_ = 0.45. No conservation rate was reported in [35] for *S. kudriavzevii*. From the topology, proximity *q*_kud_ should lie between those of *S. mikatae* and *S. bayanus* and we simply set it to *q*_kud_ = 0.5. PhyloGibbs, PhyME, and EMnEM were all run with this phylogenetic tree. EMnEM requires branch lengths in terms of the number of substitutions per site, and we used *q* = *e* ^−*n*^ to determine the number of substitutions *n* in terms of the proximity *q*.

For reference we again give the command lines that we used in running the algorithms on the 200 genes with documented sites. For PhyloGibbs with phylogeny we used –D 1 –T 0.35 –m 10 –N 3 –F bgfile –I 3,3,3 –E 0.01 –f infile –L (cer:0.8,par:0.8,mik:0.58,kud:0.5, bay:0.45). Here bgfile is a fasta file with all *S. cerevisiae* intergenic sequences from which the background model is constructed. The setting –T 0.35 sets the pseudocount of the WM prior to 0.35 to account for the fact that TFs in *S. cerevisiae* generally have higher information scores than random WMs. We ensured that the fasta header for each sequence identified the name of the species from which it derived. Finally, the setting –E 0.01 instructs PhyloGibbs to report sites with probabilities as low as 0.01 (instead of the default 0.05). For EMnEM the relevant parameters in the control file were –w 10 –e 3.0 –t 0.05 –b 1 –n 3 –u 1 –r 0 –m 0. EMnEM was also provided with the phylogenetic tree as described above. It uses ClustalW alignments [30] of the upstream regions. For PhyME we used –N 1 infile blkfile –w 10 –nmotifs 3 –revcompW –ot 0.05 –nsites 3 –niter 50 –nseediter 10 –b –K 5 –pf phylogenytree.txt –tree. PhyME uses MLAGAN [29] for its alignments and parses these in its own specific way. The results of this parse are in the blkfile. For PhyloGibbs without phylogeny we used –D 0 –T 0.35 –m 10 –N 3 –F bgfile –I 10,10,10 –E 0.01 –f infile; for WGibbs, –PBernoulli infile 10,10,10 10,10,10 –Z –n; and for MEME, infile –dna –mod tcm –w 10 –nmotifs 3 –wg 1000000 –nomatrim –maxiter 500 –maxsize 1000000 –revcomp –bfile bgfile.

We should point out that the performances of the different algorithms may vary as one varies parameter settings. We experimented with different parameter settings for each of the algorithms but none substantially changed the results shown in Figure 6. For all parameter settings that we tested, PhyloGibbs with phylogeny outperformed all other algorithms. We did notice that PhyME and EMnEM were much more sensitive to the phylogenetic tree than PhyloGibbs was. PhyME showed best performance with the tree that we show here. EMnEM could be made to perform better than the non-phylo algorithms by using a tree with shorter branch lengths.

The specificity-versus-sensitivity plots in the left panel of Figure 6 were obtained almost identically to what was described for the synthetic data. The only difference was that, instead of counting the precise number of bases in predicted sites overlapping bases in true sites, we considered any predicted site to “hit” a true site if it overlapped the true site by at least 5 bp (half of the predicted site's length). We did this because the precise extent of the known sites seems often poorly defined: Typically one finds that different sites that are annotated for the same TF in [26] can have widths that vary substantially.

For the right panel of Figure 6 we show, as in the right panel of Figure 5, the predicted specificities *s_p_* of the different sublists on the horizontal axis, but on the vertical axis we show the ratio *s_r_*/*s_p_* between the measured specificity *s_r_* on documented sites and the specificity *s_p_* that the algorithm predicts.

### Regulatory code.

We used version 24 of the regulatory code from [27]. In particular we used the set of “final motifs,” and the highest-confidence binding sites (binding with *p* < 0.001 and conserved in at least two other yeasts) based on these motifs. The former consists of a file (Final_InTableS2_v24.motifs) with 102 WMs for 102 TFs, and the latter consists of a file (IGR_v24.3.p001b.GFF) with the genomic locations of 3,353 predicted binding sites for these 102 WMs. From the set of WMs we selected the 45 WMs for which there were at least three annotated sites and at most 25. The file Final_InTableS2_v24.motifs notes which WMs are based solely on sites/consensus reported in the literature. Among the 45 WMs that we selected there were 21 that were literature based, i.e., no significant motif was found by the computational methods employed in [27]—these are the WMs shown in Table 1. For each of the 45 selected WMs we collected all intergenic regions with annotated binding sites and their orthologs in the four other sensu stricto species, and aligned them with Dialign as described above. We then ran PhyloGibbs twice on each of the 45 collections of intergenic regions. For a WM with a total of *S* annotated sites in [27] and a motif width of *l* in [27] we used the following command line settings for the two runs: (1) –D 1 –L (cer:0.8, par:0.8, mik:0.58, kud:0.5, bay:0.45) –T 0.25 –m *l* –N 3 –F bgfile –I *S,S,S* –f infile and (2) –D 1 –L (cer:0.8, par:0.8, mik:0.58, kud:0.5, bay:0.45) –T 0.25 –m 15 –N 3 –F bgfile –I *S,S,S* –f infile. That is, we used both the annotated binding site width and a default width of 15. Since there generally are binding sites for multiple WMs in the set of intergenic regions, we let PhyloGibbs search for three different motifs with a total number of sites equaling three times the number of annotated sites in [27].

To compare the results of PhyloGibbs with those of [27] we compared the configurations of binding sites that PhyloGibbs reported with all the motifs reported in [27]. In particular, for each motif that PhyloGibbs reports it outputs two alignments of predicted sites. One alignment consists of the sequences that have a common color in the reference configuration C*. The other consists of the time-averaged alignment of sequences that associate with this reference color during tracking. For each WM *w* in the file Final_InTableS2_v24.motifs we multiplied the WM entries *w*_α*i*_ by the total number of sites *S* annotated for the WM to obtain an alignment *m* of all the binding sites, with *m*_*αi*_ = *w*_*αi*_ *S* the number of sites that have base *α* at position *i*. For each pair of one such alignment from [27] and a reported alignment from PhyloGibbs we calculated the probability that both alignments were drawn from a common WM.

Let *n* be one of the alignments of sites reported by PhyloGibbs and *m* be an alignment of sites from [27], with *n*_α*i*_ and *m*_α*i*_ being the number of times base α occurs at position *i* of alignments *n* and *m,* respectively. We now calculate the probability that these two alignment were sampled from a common WM, taking into account the possibility that *n* and *m* may be shifted or reverse-complemented with respect to each other. Assume *n* has width *l_n_* and *m* has width *l_m_* and assume an alignment of *n* and *m* in which *n* is shifted *k* positions to the right with respect to *m*. The total number of times *t*_*αi*_ that base α occurs at position *i* in this joint alignment is given by (1) *t*_α*i*_ = *m*_α*i*_ when 1 ≤ *i* ≤ *k,* (2) *t*_*αi*_ = *m*_*αi*_ + *n*_*α*(*i − k)*_ when (*k* + 1) ≤ *i* ≤ *l_m_,* and (3) *t*_*αi*_ = *n*_*α*(*i − k)*_ when *l_m_* + 1 ≤ *i* ≤ *l_n_* + *k*. The probability to draw this joint alignment *t* from a WM is


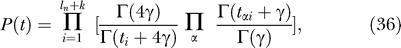


where γ is the pseudocount of the prior over WM space and *t_i_* is the total number of bases in column *i* of the joint alignment *t*. We here use the uniform prior γ = 1. The probability to draw *n* and *m* from two separate WMs is similarly given by *P*(*n*)*P*(*m*) with each factor given by the same equation 36. Thus, the posterior probability *P*(*t*|*n,m*) that *n* and *m,* forming joint alignment *t,* were drawn from a common WM is given by


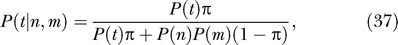


where π is the prior probability. For each alignment *n* that PhyloGibbs reported there were 102 alignments *m* from [27] (one for each TF), and for each of those we considered all relative shifts and reverse-complement combinations in which at least 4 bp overlapped between *m* and *n*. For alignments of length *l_n_* and *l_m_* there are 2(*l_n_* + *l_m_* − 8) such combinations. We set π such that the prior probability was 1/2 that any of these shift/strand combinations from any of the 102 WMs *m* gave an alignment *t* that was sampled from a common WM. That is, we set


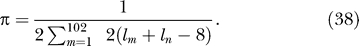


Finally, for each combination *n* and *m* we calculated the maximum of *P*(*t*|*n,m*) over all 2(*l_n_* + *l_m_* − 8) shift/strand combinations *t* to obtain


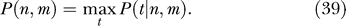


In the Dataset S2 we show all *P*(*n,m*) that are larger than 1/4.

In addition, for each combination of a reported motif from PhyloGibbs and a TF with annotated sites in [27] we calculated the fraction of sites in the motif that overlapped (by at least 4 bp) a site annotated for that TF in [27]. For the motifs reported in tracking we again weighed each site by its posterior probability in calculating this fraction. Dataset S2 shows all combinations of reported motifs and TFs for which this fraction is non-zero.

The results in Tables 1 and 2 were calculated as follows. After running PhyloGibbs on the set of upstream region alignments for one of the 45 TFs, we analyzed all three motifs that PhyloGibbs reported and determined which one best matched the motif *m* reported for the TF in [27]. For each motif we obtained the alignment of sequences *n_r_* reported in the reference configuration C* and the time-averaged alignment *n_t_* obtained for this motif through tracking, and calculated the probabilities *P*(*n_r_ ,m*) and *P*(*n_t_ ,m*) that these alignments were sampled from the same WM as the alignment *m* of sites reported in [27]. We also calculated the fractions *f*(*n_r_ ,m*) and *f*(*n_t_ ,m*) of sites in *n_r_* and *n_t_* that overlapped sites annotated for the motif *m* in [27]. The total score *s*(*n*) of the motif was simply given by the sum *s*(*n*) = *P*(*n_r_ ,m*) + *P*(*n_t_ ,m*) + *f*(*n_r_ ,m*) + *f*(*n_t_ ,m*). For the motif (out of three) that maximizes *s*(*n*), Tables 1 and 2 show *P*(*n_r_ ,m*) in the second column and *P*(*n_t_ ,m*) in the third column. The fourth column shows the total number of sites |*n_r_*| in this motif (color) in reference configuration C* and the total number of those *f*(*n_r_ ,m*)|*n_r_*| that overlap sites annotated for *m* in [27]. The fifth column shows the same statistics for the tracked set of sites *n_t_*, where again each site is weighed by its posterior probability. That is, |*n_t_*| is the sum of the posterior probabilities of the sites in this motif. Finally, the sixth column shows the total number of sites |*m*| that were annotated for motif *m* in [27].

For 11 TFs we gathered sets of target genes from the literature, collected their orthologs from the other sensu stricto species, obtained multiple alignments with Dialign, and ran PhyloGibbs on these sets of multiple alignments. The following command line options were used for all these runs: –D 1 –T 0.25 –a 300 –S 300 –L (cer:0.8, par:0.8, mik:0.58, kud:0.5, bay:0.45) –N 3 –F bgfile –f infile. A summary of the results of these runs, and the remaining parameter settings used, are shown in Table 3.

**Table 3:**
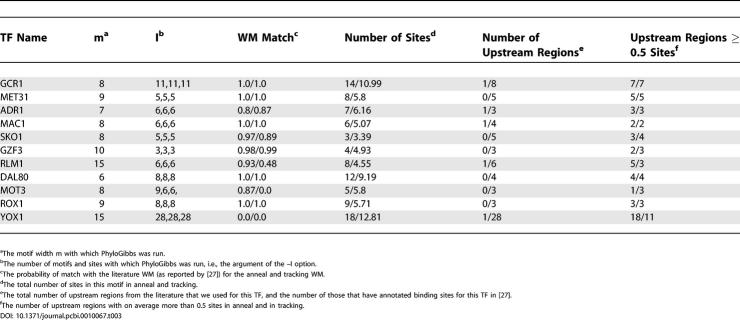
Results of PhyloGibbs on Multiple Alignments of Upstream Regions Taken from the Literature

Detailed results, and the locations of all the binding sites newly identified in these runs, can be found in Datasets S4 and S5.

## Supporting Information

Dataset S1 Predicted Sites on Genes with Sites in SCPD This file lists all sites with posterior probability 0.05 or higher that PhyloGibbs predicted on the upstream regions of the genes that have one or more binding sites annotated in the SCPD [26]. The sites are ordered first by posterior probability, then by the name of the open reading frame (ORF), and finally by the number of the motif in which the site occurred. An example line from the file is “YPR191W (−175, −166) rev 0.97 taagaCGGGGCGGGCcttct 3.” The first column shows the name of the ORF, the second column shows the location of the site relative to the ATG of the ORF, the third column shows the strand on which the site occurs, the fourth column shows the posterior probability of the site, and the fifth column shows the sequence of the site (in capitals) plus five bases to the left and right of the site.
(141 KB TXT)

Dataset S2 Comparison of the PhyloGibbs Predictions with Those of Harbison et al. [27] This file summarizes the comparisons of the results of PhyloGibbs on the data from [27] with those reported in [27].
(99 KB TXT)

Dataset S3 All Predicted Sites on the Data from [27] This file contains all binding sites with posterior probability at least 0.05 that PhyloGibbs predicted for the 45 TFs with between three and 25 sites annotated in [27]. For each TF PhyloGibbs was run on the five-species multiple alignments of all upstream regions with sites annotated in [27] and asked to predict three motifs. In this file we show only the predictions for the motif that best matched the motif reported in [27]. The format of the lines is very similar to that of the lines in Dataset S1. Example for a predicted site for TF ADR1: ADR1 YKL016C (−388,–383) fwd 0.58 tacTCCAATatt harb_lit. The site occurs 388 to 383 bases upstream of the ATG of the ORF YKL016C. It occurs on the forward strand of the genome and has a posterior probability 0.58. The fifth column shows the sequence of the site in capitals plus half a site length to the left and right. Finally, the last column shows if [27] found the WM of this TF by computational means or if they simply copied the motif reported in the literature.
(42 KB TXT)

Dataset S4 Co-Regulated Gene Sets Gathered from the Literature For 11 TFs we gathered lists of genes that are known to be regulated by the TF from the literature. This file gives the list of ORF names of these genes for each of the 11 TFs. Example: DAL80 YKR034W YIR032C YDL210W YFL021W. This line shows that the TF DAL80 is reported in the literature to regulate the ORFs YKR034W, YIR032C, YDL210W, and YFL021W.
(10 KB TXT)

Dataset S5 Predicted Sites for the Literature Gene Sets This file has the same format as Dataset S3 and shows all predicted sites for the 11 TFs in the upstream regions of the genes in Dataset S4. Example: GCR1 YAL038W (−263, −256) rev 0.81 ttttAGGAAGACacta. This example shows a predicted site for TF GCR1 which occurs from 263 to 256 bases upstream of the ATG of YAL038W, occurs on the negative strand, and has posterior probability 0.81.
(9 KB TXT)

Figure S1 Analog of Figure 3 with Yeast WMs and Proximities Results analogous to those shown in Figure 3 but with “real” WMs representing known binding specificities of yeast TFs, and using a phylogenetic tree with branch lengths proportional of those of the *Saccharomyces* sensu stricto species.
(62 KB PDF)

Figure S2 Analog of Figure 5 with Yeast WMs and Proximities Results analogous to those shown in Figure 5 but with “real” WMs representing known binding specificities of yeast TFs, and using the phylogenetic tree of the *Saccharomyces* sensu stricto species.
(110 KB PDF)

Figure S3 Rescaled Specificity/Sensitivity of the Predictions on SCPD Genes Results as in the left panel of Figure 6 but with specificities rescaled assuming that only 40% of all true binding sites are documented.
(60 KB PDF)

Table S1 Accuracy of the WM Polynomial Approximation This table shows a comparison of the exact WM integrals with the monomial approximation that our algorithm employs.
(7 KB PDF)

## Abbreviations

EM: expectation maximization
ORF: open reading frame
SCPD: Promoter Database of *Saccharomyces cerevisiae*
TF: transcription factor
WM: weight matrix
