# Supplementary material for: PhyloGibbs: A Gibbs Sampling Motif Finder That Incorporates Phylogeny
Source: PLoS Comput Biol. 2005 Dec 9;1(7):e67. doi: 10.1371/journal.pcbi.0010067 (PMC1309704; doi:10.1371/journal.pcbi.0010067)
Supplement: Supplementary file 1 [file pcbi.0010067.sg001.pdf]

Figure S1: Analog of Figure 3 with yeast WMs and proximities

Rahul Siddharthan   Eric D. Siggia   Erik van Nimwegen

October 28, 2005

We created synthetic data-sets analogous to those shown in the lower-right panel of Fig. 3 in the main text, but using "real" WMs representing the binding specificities of yeast TFs, and using branch lengths in the phylogenetic tree that are proportional to those for the *Saccharomyces sensu stricto* species. Formally, we took each of the 102 WMs inferred in [1] and made them all uniform width $w = 10$. WMs wider than $w = 10$ were cropped symmetrically, and WMs shorter than $w = 10$ were padded with random columns $w_a = w_c = w_g = w_t = 1/4$. For each data-set we chose one of the 102 WMs at random and embedded $s = 4$ sites in a random sequence of length $L = 500$. We then created $S = 5$ descendant sequences at phylogenetic distances that are proportional to those of the 5 *Saccharomyces sensu stricto* species. Those proximities are given by $q_{\mathrm{cer}} = 0.8$, $q_{\mathrm{par}} = 0.8$, $q_{\mathrm{mik}} = 0.58$, $q_{\mathrm{kud}} = 0.5$, and $q_{\mathrm{bay}} = 0.45$. The proximities are related to branch lengths by the equality

$$q_s = e^{-b_s}. \tag{1}$$

Using this we first transform all proximities q_s into branch lengths. For the synthetic data we multiply each branch length with a factor λ, with λ ranging from $\lambda = 0.25$ to $\lambda = 4$. Finally, we transform the branch lengths back to proximities using (1). At $\lambda = 0.25$ this leads to proximities $q_1 = 0.948$, $q_2 = 0.948$, $q_3 = 0.873$, $q_4 = 0.841$, $q_5 = 0.819$. At $\lambda = 1$ the proximities of course match the *Saccharomyces* proximities, and at $\lambda = 4$ the proximities are $q_1 = 0.41$, $q_2 = 0.41$, $q_3 = 0.11$, $q_4 = 0.06$, $q_5 = 0.04$. In the figure we use the geometric mean of the 5 proximities as an indication of the "average" proximity for each data-set. The performance of the algorithms is measured in the same way as for Fig. 3 in the main text.

As the results show, the performance of PhyloGibbs (with phylogeny) on this data is quantitatively close to the performance on the data in the upper-right panel if Fig. 3. The nonphylo algorithms perform even more poorly on this data than on the data in the upper-right panel of Fig. 3 in the main text.

We also tested if the nonphylo algorithms would perform better if they were run on a single sequence with $s = 4$ embedded sites instead of on all $S = 5$ orthologues (containig $Ss = 20$ sites in total). We ran MEME and WGibbs on single sequences, asking them to find 4 sites of length $w = 10$. Both algorithms performed even more poorly in this test. The sites that MEME predicted had an average overlap of 0.077 ± 0.011 with the true sites, and WGibbs had an average overlap of 0.096 ± 0.013. These performances are more than twice as low as the performance when running on all $S = 5$ orthologues. In fact, the algorithms do not perform statistically better than what would be expected by randomly placing windows: the sites cover $4 * 10 = 40$ of the 500 bases, which corresponds to 8% of the input sequence. It thus appears that, at least for yeast WMs, the nonphylo algorithms clearly benefit from using the orthologous sequences, even if they treat them as independent.

References

[1] Harbison CT, Gordon DB, Lee TI, Rinaldi NJ, Macisaac KD, et al. (2004) Transcriptional regulatory code of a eukaryotic genome. Nature 431:99–104.

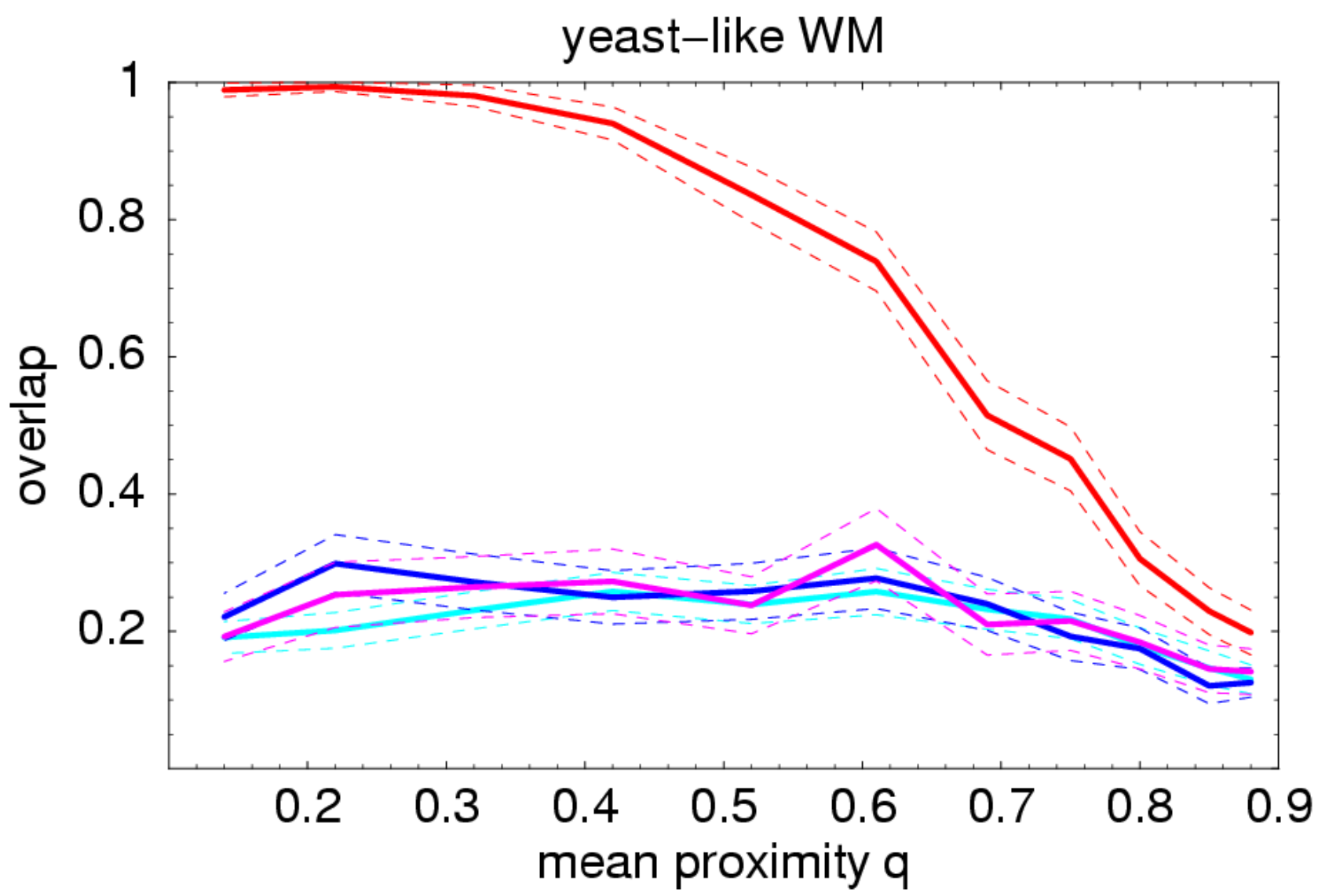

Results of a test analogous to the one shown in Fig. 3 in the main text but with "real" WMs from yeast, and using phylogenetic distances proportional to the phylogenetic distances of the *Saccharomyces* species. The overlap between the embedded and predicted sites at different average proximities is shown for PhyloGibbs with phylogeny (red), PhyloGibbs in nonphylo mode (light blue), WGibbs (dark blue), and MEME (pink).
