# Supplementary material for: PhyloGibbs: A Gibbs Sampling Motif Finder That Incorporates Phylogeny
Source: PLoS Comput Biol. 2005 Dec 9;1(7):e67. doi: 10.1371/journal.pcbi.0010067 (PMC1309704; doi:10.1371/journal.pcbi.0010067)
Supplement: Supplementary file 2 [file pcbi.0010067.sg002.pdf]

Figure S2: Analog of Figure 5 with yeast WMs and proximities

Rahul Siddharthan   Eric D. Siggia   Erik van Nimwegen

October 28, 2005

We created synthetic data-sets analogous to those use in Figure 5 of the main text, but again using yeast WMs and using the proximities of the *Saccharomyces sensu stricto* species. For each intergenic region we randomly picked 3 WMs from the list of 102 WMs of [1] and embedded 3 sites for each of the WMs in a random sequence of length $L = 750$. Five descendant sequences were generated at proximities $q_1 = 0.8$, $q_2 = 0.8$, $q_3 = 0.58$, $q_4 = 0.5$, $q_5 = 0.45$. All phylo algorithms were given the correct phylogenetic tree relating the descendants all other command line options were the same as for the tests in Fig. 5 of the main text, accept for the WM prior used in PhyloGibbs. A prior of `-T 0.35` was used to reflect the higher average information content of the yeast WMs. The performance was measured exactly as in Fig. 5. The left panel shows how the fraction of predicted sites that match true sites (specificity) depends on the fraction of true sites that are among the predictions (sensitivity) for PhyloGibbs (red), EMnEM (yellow), PhyME (green), PhyloGibbs without phylo (light blue), WGibbs (dark blue), and MEME (pink). Dashed lines correspond to two standard-errors. The right panel shows the ability of the different algorithms to assess their own reliability. The true specificity is shown as a function of the specificity that the algorithm predicts for the sites that it reports. The black line $y = x$ corresponds to a perfect assessment of the algorithm's reliability.

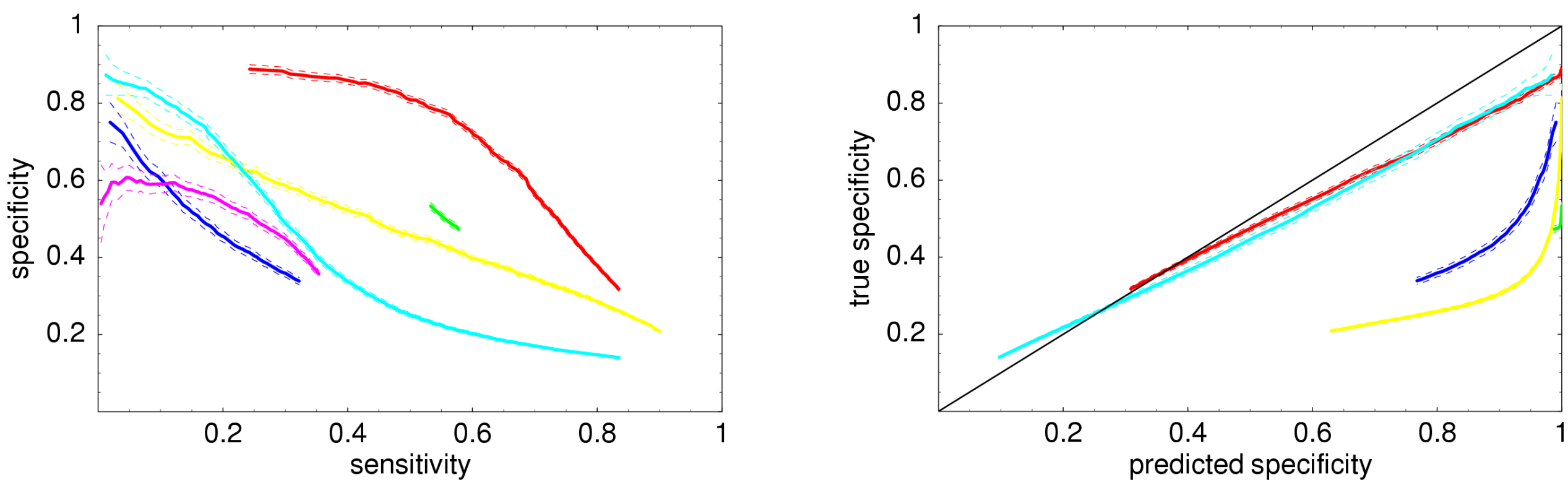

In comparison with Fig. 5 of the main text all algorithms perform significantly better. This, we believe, is mainly a result of the fact that the yeast WMs tend to have higher information scores than the random WMs used in figure 5. The phylo algorithms still clearly outperform the nonphylo algorithms although PhyloGibbs without phylogeny slightly outperforms EMnEM at low sensitivies. This is most likely a result of PhyloGibbs' tracking strategy that allows for better estimates of the posterior probabilities of the predicted sites. In contrast to Fig. 5 where EMnEM and PhyME performed equally well, PhyME outperforms EMnEM on this test. PhyloGibbs with phylogeny still clearly outperforms all other algorithms.

The right panel shows that, as in Fig. 5, all algorithms but PhyloGibbs strongly overestimate the reliability of their predictions. In this test PhyloGibbs also slightly overestimates the reliability of its predictions at high specificity. This is a result of a slight technical difference in the meaning of "posterior probability" as measured in this test, and as estimated in the tracking procedure. In the tracking procedure, a site that is slightly shifted with respect to the site in the reference configuration C^* is counted as the "same" site. Thus, the posterior probability calculated in tracking formally corresponds to the probability that the site in C^* *or a slightly shifted version* is a true site. In contrast, in

figure 5 in the main text, and in the figure above, we count a site that is shifted by (say) 2 bases with respect to the true site as only 80% correct. Therefore, if the predicted site in C^* was shifted by a single base with respect to the true site, then even if it tracks 100% of the time, it will only correspond to a site that is 90% correct. This is also the reason that the specificity saturates at 90% as opposed to 100%. If we had used a more lenient definition of a “match” between true and predicted sites the specificities of PhyloGibbs would be increased and the curves in the right-panel would lie above the line $x = y$ (data not shown).

References

[1] Harbison CT, Gordon DB, Lee TI, Rinaldi NJ, Macisaac KD, et al. (2004) Transcriptional regulatory code of a eukaryotic genome. Nature 431:99–104.
