# Supplementary material for: PhyloGibbs: A Gibbs Sampling Motif Finder That Incorporates Phylogeny
Source: PLoS Comput Biol. 2005 Dec 9;1(7):e67. doi: 10.1371/journal.pcbi.0010067 (PMC1309704; doi:10.1371/journal.pcbi.0010067)
Supplement: Supplementary file 3 [file pcbi.0010067.sg003.pdf]

Figure S3: Rescaled specificity/sensitivity of the predictions on SCPD genes

Rahul Siddharthan   Eric D. Siggia   Erik van Nimwegen

October 28, 2005

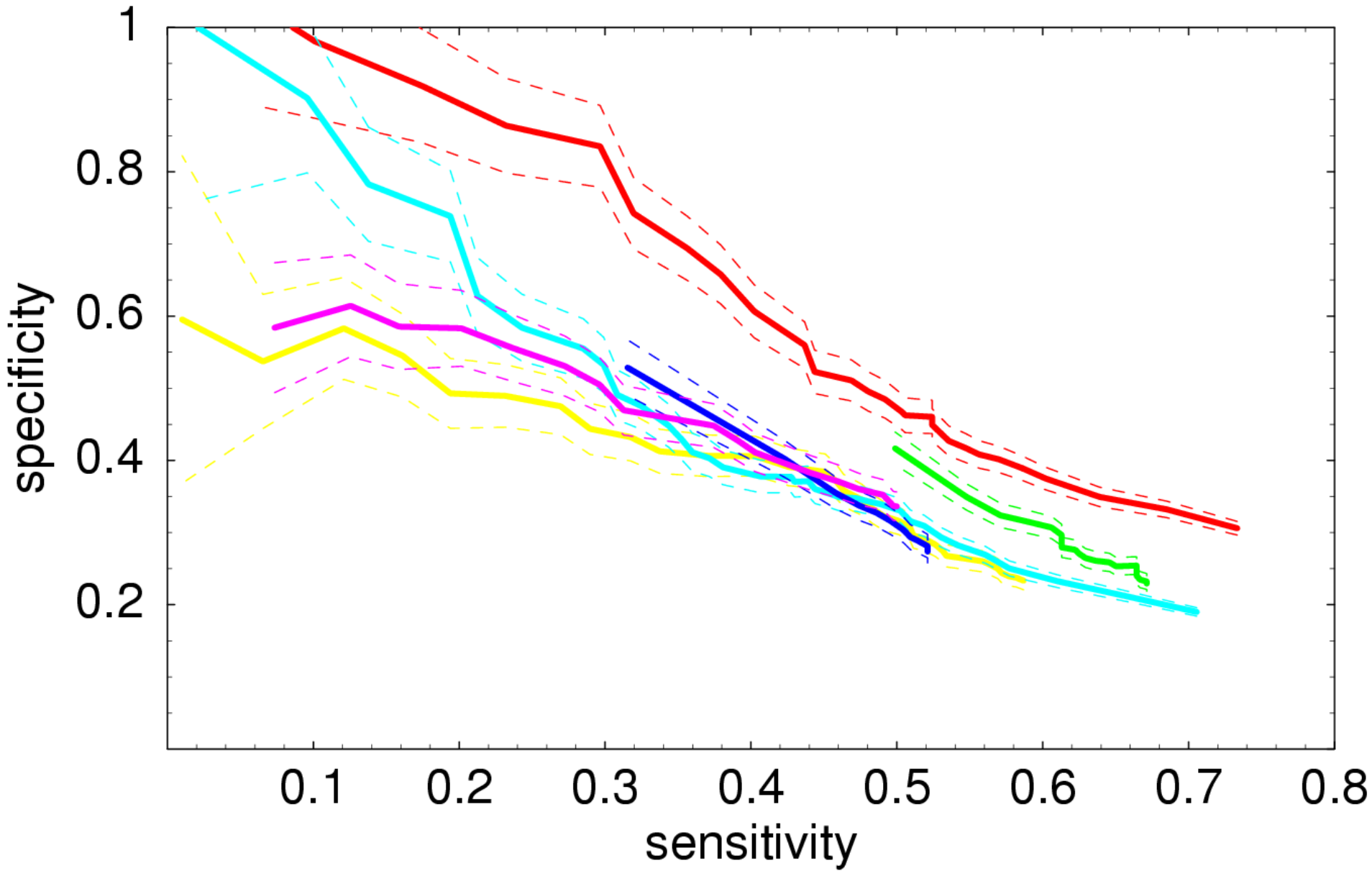

Performance of several motif finding algorithms on 200 alignments of orthologous intergenic regions from 5 *Saccharomyces* species containing documented binding sites. Estimated specificities are shown as a function of sensitivity for PhyloGibbs with phylogeny (red), PhyME (green), EMnEM (yellow), PhyloGibbs with phylogeny (light blue), WGibbs (dark blue), and MEME (pink). This figure is the same as the left panel of Fig. 6 in the main text, accept for the fact that all specificities on the vertical axis have be scaled up by a factor of 2.5. We conservatively estimate that there are 2.5 times as many real sites in the data as there are documented sites. Therefore, the specificities in this figure are more likely to reflect the true specificities of the various algorithms on the real data.
