# Supplementary material for: PhyloGibbs: A Gibbs Sampling Motif Finder That Incorporates Phylogeny
Source: PLoS Comput Biol. 2005 Dec 9;1(7):e67. doi: 10.1371/journal.pcbi.0010067 (PMC1309704; doi:10.1371/journal.pcbi.0010067)
Supplement: Supplementary file 4 [file pcbi.0010067.st001.pdf]

Supporting Table S1: Accuracy of the WM-polynomial Approximation

Rahul Siddharthan Eric D. Siggia Erik van Nimwegen

October 28, 2005

Initial motif + added bases	Exact q=0.2	Approx q=0.2	Exact q=0.5	Approx q=0.5	Exact q=0.8	Approx q=0.8
AAAA	4.480	4.480	1.782	1.782	1.163	1.163
+ A	10.767	10.767	3.657	3.657	2.033	2.033
+ AA	28.014	27.910	8.634	8.524	4.322	4.288
+ AAA	77.121	76.505	22.238	21.649	10.334	10.170
+ AAAA	221.581	218.915	60.852	58.499	26.747	26.135
+ AAAAA	658.449	648.170	174.199	165.669	73.317	71.219
+ AAAAAA	2010.814	1973.182	516.480	486.782	209.939	202.965
+ AAAAAAA	6281.266	6146.959	1575.074	1473.396	622.189	599.239
+ AAAAAAAA	19998.835	19525.479	4916.044	4570.058	1896.056	1820.599
AAAA	4.480	4.480	1.782	1.782	1.163	1.163
+ A	10.767	10.767	3.657	3.657	2.033	2.033
+ AT	5.018	5.052	1.998	2.035	1.270	1.281
+ ATA	11.645	11.712	4.100	4.149	2.317	2.327
+ ATAT	8.807	8.925	3.516	3.641	2.204	2.243
+ ATATA	19.956	20.193	7.198	7.396	4.114	4.165
+ ATATAT	19.042	19.400	7.623	8.000	4.738	4.858
+ ATATATA	42.412	43.139	15.568	16.210	8.966	9.145
+ ATATATAT	46.564	47.646	18.682	19.826	11.548	11.916
AACG	0.601	0.601	0.576	0.576	0.549	0.549
+ A	0.880	0.880	0.779	0.779	0.673	0.673
+ AA	1.546	1.542	1.309	1.299	1.064	1.057
+ AAT	0.623	0.623	0.542	0.541	0.458	0.457
+ AATA	1.123	1.120	0.946	0.938	0.762	0.756
+ AATAA	2.233	2.222	1.839	1.811	1.430	1.411
+ AATAAC	1.381	1.375	1.159	1.144	0.928	0.918
+ AATAACA	2.748	2.731	2.263	2.221	1.760	1.732
+ AATAACAA	5.841	5.796	4.742	4.629	3.602	3.528

Exact values of the WM integral, and our monomial approximation, when a series of independent bases is added successively to an aligned set of four bases descended from a common ancestor with proximity q. Even when eight bases are added to the original four, the errors are relatively small.
